# Epithelial heparan sulfate regulates Sonic Hedgehog signaling in lung development

**DOI:** 10.1371/journal.pgen.1006992

**Published:** 2017-08-31

**Authors:** Hua He, Meina Huang, Shenfei Sun, Yihui Wu, Xinhua Lin

**Affiliations:** 1 State Key Laboratory of Membrane Biology, Institute of Zoology, Chinese Academy of Sciences, Beijing, China; 2 University of Chinese Academy of Sciences, Beijing, China; 3 State Key Laboratory of Genetic Engineering, Institute of Genetics, Collaborative Innovation Center of Genetics and Development, School of Life Sciences, Fudan University, Shanghai, China; 4 Division of Developmental Biology, Cincinnati Children’s Hospital Medical Center, Cincinnati, OH, United States; University of Wisconsin, UNITED STATES

## Abstract

The tree-like structure of the mammalian lung is generated from branching morphogenesis, a reiterative process that is precisely regulated by numerous factors. How the cell surface and extra cellular matrix (ECM) molecules regulate this process is still poorly understood. Herein, we show that epithelial deletion of Heparan Sulfate (HS) synthetase *Ext1* resulted in expanded branching tips and reduced branching number, associated with several mesenchymal developmental defects. We further demonstrate an expanded *Fgf10* expression and increased FGF signaling activity in *Ext1* mutant lungs, suggesting a cell non-autonomous mechanism. Consistent with this, we observed reduced levels of SHH signaling which is responsible for suppressing *Fgf10* expression. Moreover, reactivating SHH signaling in mutant lungs rescued the tip dilation phenotype and attenuated FGF signaling. Importantly, the reduced SHH signaling activity did not appear to be caused by decreased *Shh* expression or protein stability; instead, biologically active form of SHH proteins were reduced in both the *Ext1* mutant epithelium and surrounding wild type mesenchymal cells. Together, our study highlights the epithelial HS as a key player for dictating SHH signaling critical for lung morphogenesis.

## Author summary

Defective development of the respiratory system leads to congenital or postnatal respiratory disorders. Although it is well established that lung morphogenesis is guided by interactions of multiple morphogen signaling pathways in epithelium and mesenchyme, how their regulatory functions are controlled by cell surface and extra cellular matrix (ECM) molecules is still largely unknown. Heparan Sulfate (HS) glycosaminoglycans are widely presented on the cell surface and in the extracellular matrix depending on the attached core proteins, and are thought to be important for transducing FGF signaling. The function of HS during lung development has not been studied *in vivo*. Here, using mouse genetics, we demonstrate that epithelial HS biosynthesis is critically required for lung branching morphogenesis. We reveal an unexpected role of epithelial HS in restricting FGF signaling. We show that depletion of epithelial HS leads to a dramatic loss of biologically active form of SHH, which is required for ensuring a proper expression of *Fgf10*. Our study demonstrates that the SHH-producing lung epithelial cells require HS to maintain the quality of the ligand at the source level and highlights epithelial HS as a critical regulator that dictates lung morphogenesis.

## Introduction

The developmental program of the mammalian lung is a multi-step and fine-tuned process. In mouse, it starts from the emergences of lung primordium that expresses the transcriptional factor Nkx2.1 at embryonic day (E) 9.5 in the foregut endoderm [1,2]. The two primary lung buds then repeatedly undergo morphogenic changes to generate a tree-like structure until E16.5 [2]. This high hierarchy process, referred to as branching morphogenesis is essential to generate numerous airways and gas-exchanging units, and is critically regulated by interactions of signaling pathways in the epithelium and mesenchyme. Early buds formation is dependent on reciprocal interactions between the lung epithelium and mesenchyme mediated by distinct signalings. The distal signaling center is marked by the expression of fibroblast growth factor (FGF) 10 in the mesenchyme. FGF10 signaling serves as a chemoattractive cue for epithelium outgrowth [1,3–5]. On the other hand, Sonic Hedgehog (SHH) produced in the epithelium is required for mesenchymal development and negatively regulates *Fgf10* expression, leading to a focal *Fgf10* expression pattern in the intertubular mesenchyme to facilitate the formation of new epithelial buds [3]. The SHH-FGF10 signaling, as well as other signaling pathways including WNTs and BMP pathways in the localized region act cooperatively to regulate the branching program[5–7].

Heparan sulfate glycosaminoglycans (HS-GAG) are presented as linear polysaccharide chains, and they are synthesized by the glycosytransferase exostosin (EXT) 1/2 complex that attaches alternating N-acetyl glucosamine (GlcNAc) and D-glucuronic acid (GlcA) units to the growing chain [8,9]. The HS chains covalently attach to core proteins to form Heparan Sulfate Proteoglycans (HSPGs). HS are presented both on the cell surface and in the extracellular matrix, depending on their core protein localizations [10,11]. Due to its conserved negatively charged sugar motif, HS chains exhibit a wide spectrum of binding affinity to growth factors demonstrated in invertebrates and vertebrates. In *Drosophila*, it has been shown that signaling activities of Hedgehog (Hh), FGFs and Wingless (Wg) are regulated by both HS chains and core proteins [11–13]. Various models have been proposed for HS involvement in morphogen movement, ligand distribution, and intracellular trafficking [11]. While mouse embryos lacking *Ext1* failed to gastrulate [14], it is still incompletely understood how widely the proposed mechanisms are applicable to mammalian organogenesis.

Despite the critical roles of HS in the development of mammalian organs, especially those generated from branching morphogenesis [15–20], limited studies have unveiled the role of HS in the context of lung development. Mice deficient in N-deacetylase/N-sulfotransferase-1 (NDST1) had disrupted BMP signaling and displayed a lung atelectasis associated with differentiation defects [21]. Studies using *ex-vivo* organ culture demonstrated that O-sulfated HS in the developing lung epithelium is required for FGF10 binding [22,23]. However, mice lacking Hs6st1 display postnatal changes in lung with no major organogenesis defects [24]. These results indicated a spatial-temporal regulation of lung development by HS sulfation patterns. Despite the diverse mechanisms, there is still a lack of genetic evidence for understanding the role of HS in mammalian lung development. Therefore, a conditional model to deplete the HS chains in all HSPGs would provide new insights into the general roles of HS in mammalian lung development.

In the present study, using a *Shh*^*cre*^ mouse line, we conditionally ablated *Ext1* gene in the developing mouse lung epithelium. We demonstrated that epithelial HS is required for lung branching morphogenesis by maintaining SHH signaling activity. Decreased SHH signaling activity in mutant embryos leads to an expanded and elevated *Fgf10* expression, which is likely to contribute to the dilated branching tips. We show that the production of biologically active form of SHH was reduced after loss of HS in the epithelium. Thus, these data highlight the crucial requirement of HS in SHH-producing epithelial cells to maintain SHH signaling activity, and reveal the importance of HS in lung morphogenesis.

## Results

### Epithelial loss of *Ext1* disrupts HS synthesis and lung morphogenesis

**Fig 1:**
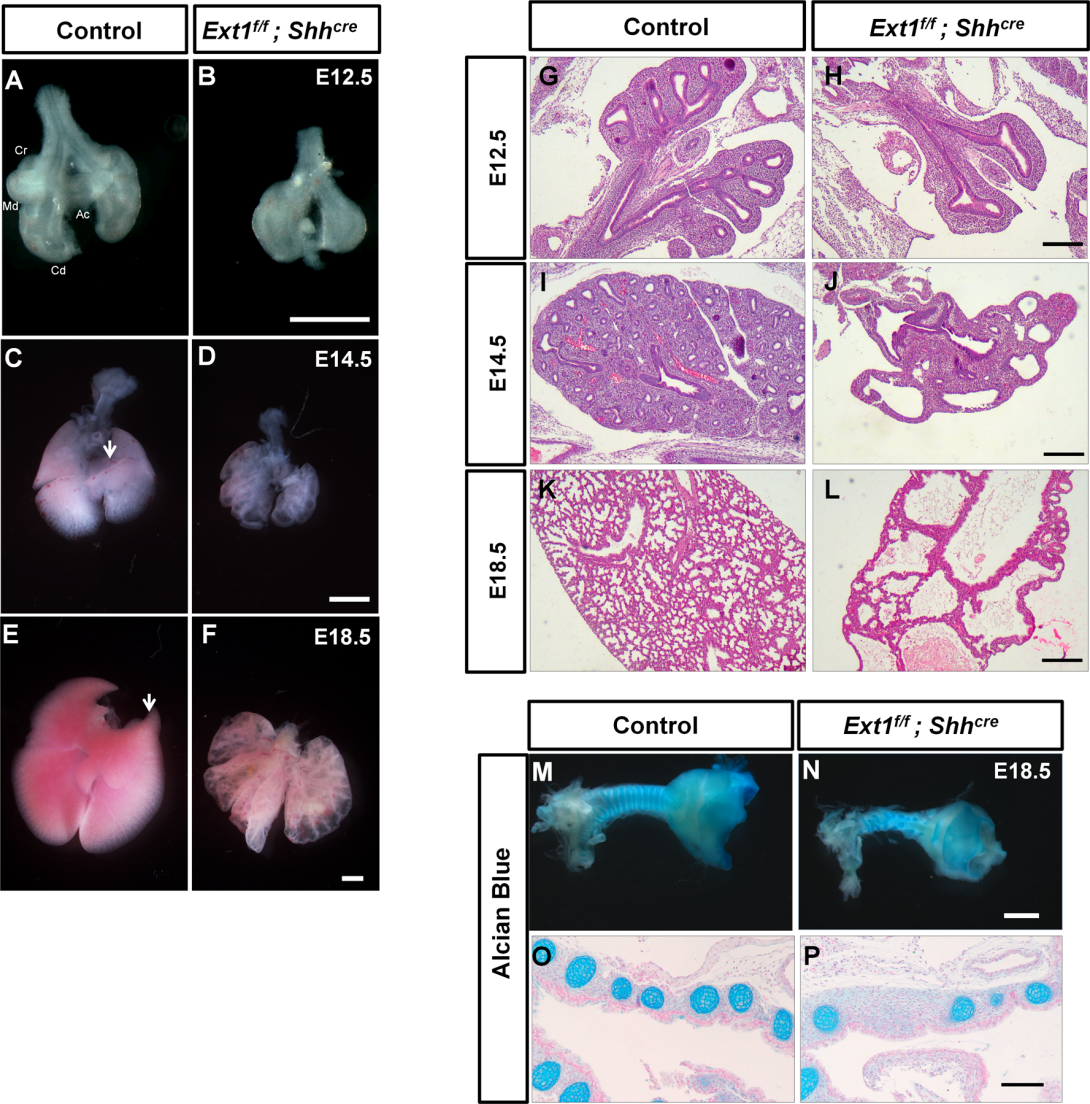
Loss of *Ext1* in the lung epithelium abrogates branching morphogenesis. (A-F) The representative gross morphology of control lungs (A, C and E) and *Ext1*^*f/f*^*; Shh*^*cre*^ mutant lungs (B, D and F) at the indicated developmental stages (E12.5, E14.5 and E18.5). The lungs from *Ext1*^*f/f*^*; Shh*^*cre*^ mutant embryos show dilated branching tips and reduced branching number in all stages. The cranial (Cr), medial (Md), caudal (Cd), and accessory (Ac) lobes were seen in control lungs (A) at E12.5, while the right lung lobes were not properly patterned in *Ext1*^*f/f*^*; Shh*^*cre*^ mutant lungs (B). The right lung lobes were specified at E14.5 and E18.5 but the Ac lobe was missing in *Ext1*^*f/f*^*; Shh*^*cre*^ mutant lungs (C-F, arrows indicate Ac lobe). (G-L) The representative histology of control lungs (G, I and K) and *Ext1*^*f/f*^*; Shh*^*cre*^ mutant lungs (H, J and L) at the indicated developmental stages (E12.5, E14.5 and E18.5). (M and N) Alcian Blue staining showing a failure of cartilage ring patterning of the trachea from *Ext1*^*f/f;*^ *Shh*^*cre*^ mutants.(O and P) The histological view of the Alcian Blue staining. Scale bars: A-F, 500μm; G-L, 200μm; M and N, 1mm;O and P, 100μm.

EXT1 protein is essential for the biosynthesis of HS chains [8]. To investigate the role of HS in lung development, we specifically ablated *Ext1* gene in the developing lung epithelium, which was achieved by a *Shh*^*cre*^ mediated recombination. *Ext1* mutant mice carry two conditional *Ext1*^flox^ alleles and a *Shh*^*cre*^ allele (*Ext1*^*f/f*^; *Shh*^*cre*^). The Cre recombinase gene in *Shh*^*cre*^ is inserted into the coding region of *Shh*, resulting in mutation of one *Shh* allele, the *Ext1*^*f/+*^; *Shh*^*cre*^ mice were therefore used as controls to eliminate the influence of *Shh* haploinsufficiency. Mutant mice were born with respiratory defects and died of breath failure shortly after birth. Analyzing the gross morphology and histology revealed that the mutant lungs were highly hypoplastic and displayed reduced branching numbers and enlarged airways at E12.5 and E14.5, with resultant cyst-like architecture in E18.5 lung (Fig 1A–1L, S1A and S1B Fig). Notably, although the lung development could be initiated, the domain branching was severely disrupted: the four right lobes were established as early as E12.5 in control lungs, while mutant lungs displayed a delayed lobe specification, and the accessory lobe was absent in later developmental stages (Fig 1A–1F). We also observed that only two lung lobes were formed in a small proportion of the mutant lungs which were most severely affected (S1C and S1D Fig). Loss of *Ext1* resulted in a failure in separation of esophagus and trachea (S1E–S1H Fig). Moreover, we observed a malformation of cartilage rings in mutant lungs (Fig 1M–1P), suggesting a cell non-autonomous requirement of *Ext1* in epithelium for mesenchymal development.

**Fig 2:**
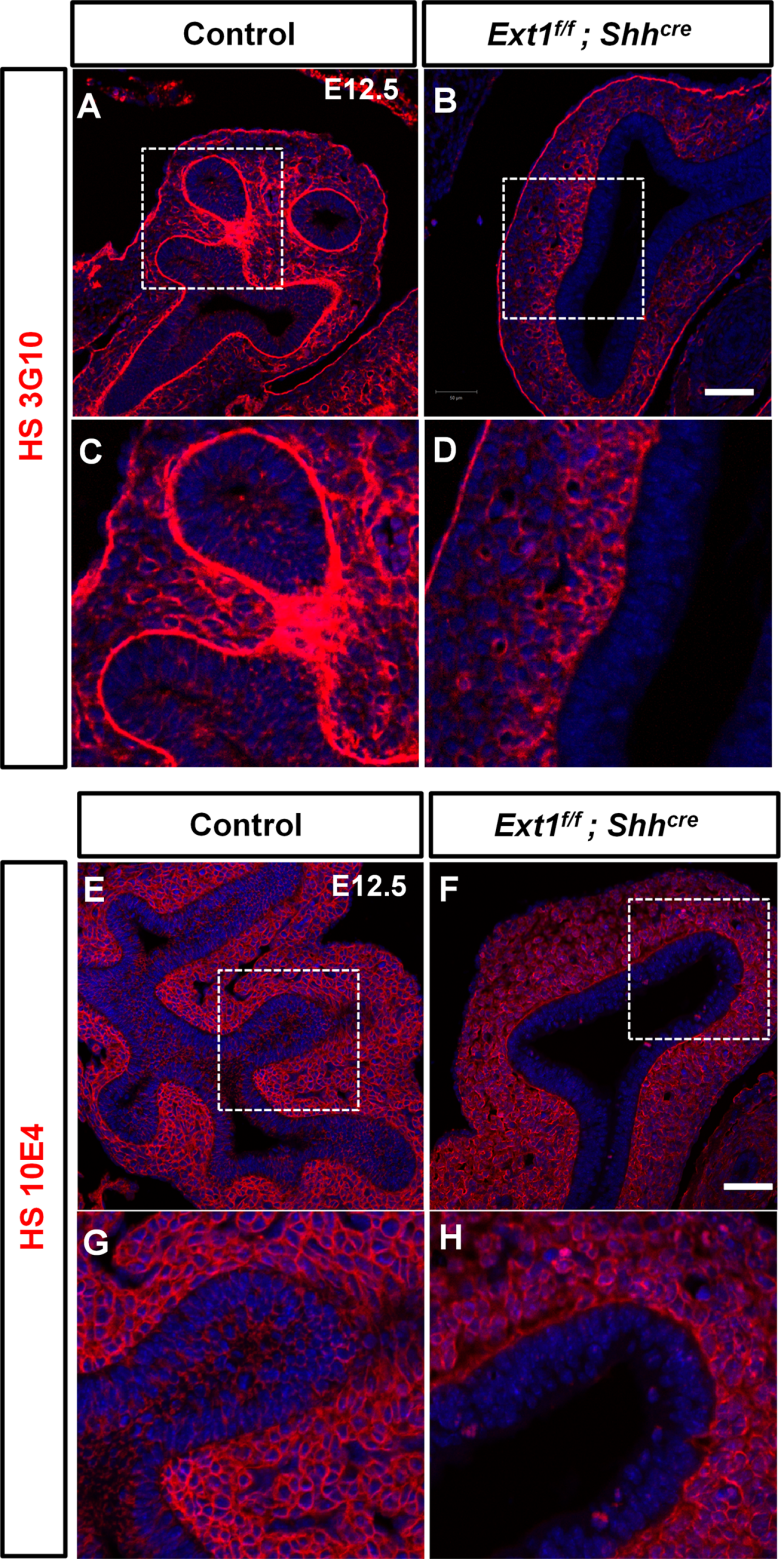
Loss of *Ext1* in the lung epithelium abrogates HS synthesis. (A-H) Immunofluorescent staining for HS (3G10) and HS (10E4) in lungs at E12.5. *Ext1* deletion abrogated the 3G10 signaling in the epithelium and basement membrane (A-D), and the 10E4 signaling in the epithelium (E-H). Scale bar: 50μm.

We next analyzed the HS distribution by immunofluorescent staining of lung sections using two common specific antibodies against HS, 3G10 and 10E4. The 3G10 antibody recognizes the neo-epitope after heparinase III digestion, which gives signals where HS is presented without providing information of HS structure. Therefore, it served as a general marker of HS. The 10E4 antibody recognizes the native HS chains of HSPG with N-sulfates and low content of O-sulfates. Consistent with previous studies[22,23], we observed that 3G10 and 10E4 signals are ubiquitously presented in the developing lung epithelium and mesenchyme. Strong 3G10 signal was found in the basement membrane where 10E4 signal is not presented (Fig 2A, 2C, 2E and 2G), indicating that HS in basement membrane is highly O-sulfated. We found that nearly all the HS 3G10 staining in the epithelial cell surface and basement membrane (Fig 2A–2D) and the HS 10E4 staining in the epithelial cell surface (Fig 2E–2H) were abolished when *Ext1* is depleted in the developing lung epithelium. These results further confirmed that deletion of *Ext1* could efficiently disrupt HS biosynthesis and that epithelial HS is critically required for lung morphogenesis.

### Epithelial HS is required for mesenchymal cell development but dispensable for epithelial cell proximal-distal patterning and differentiation

**Fig 3:**
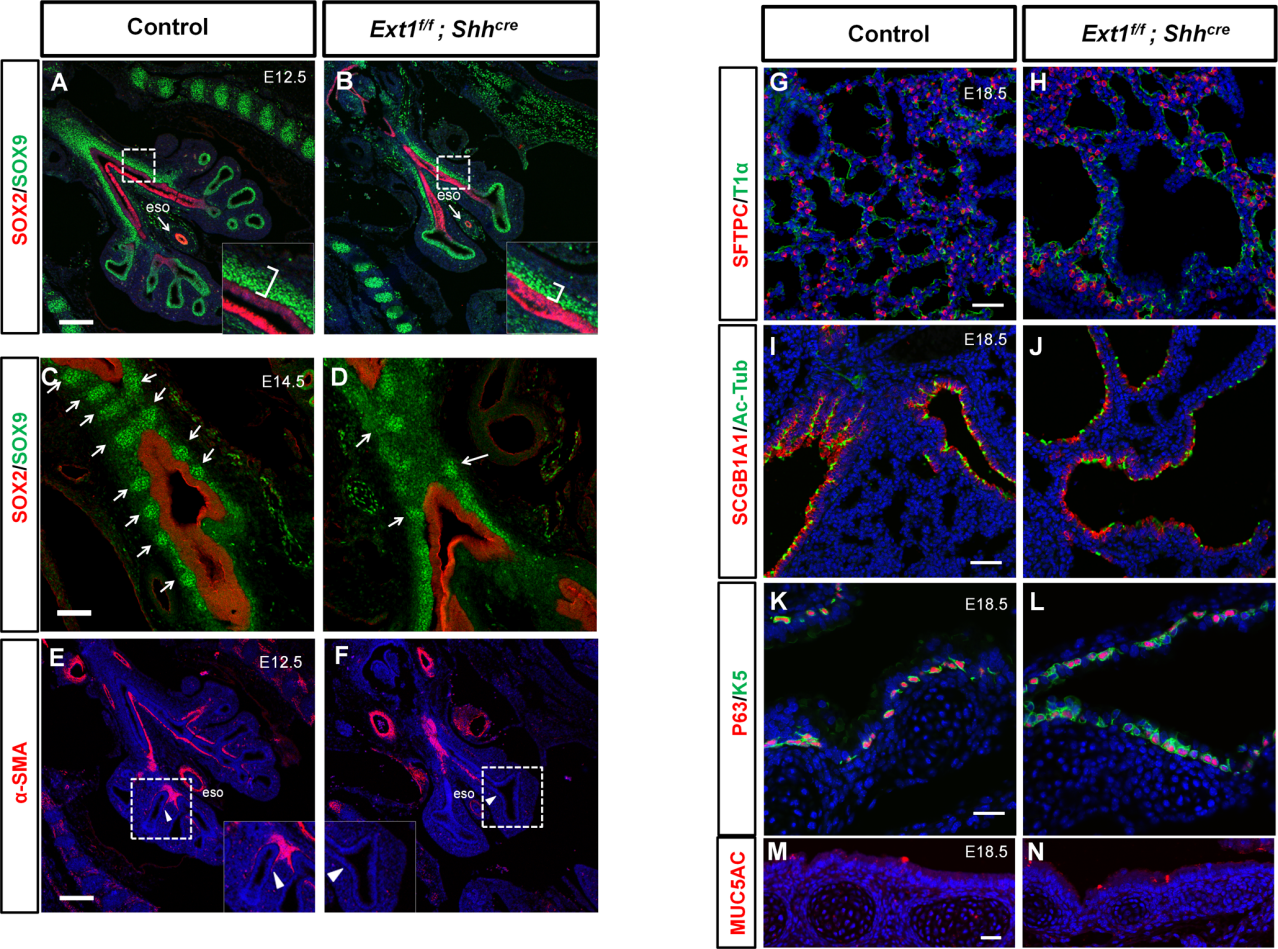
*Ext1* mutant lungs do not have epithelial differentiation and patterning defects but show maldevelopment of mesenchymal cells. (A-D) Immunofluorescent staining for SOX2/SOX9 in control lungs and mutant lungs at E12.5 and E14.5. The proximal-distal patterning was maintained in *Ext1*^*f/f*^*; Shh*^*cre*^ mutant lungs(A and B), but the mesenchymal SOX9^+^ progenitors in the tracheal region were less abundant (inserts in A and B), and SOX9^+^ mesenchymal cells were irregularly arranged at E14.5(C and D). (E and F) The α-SMA^+^ myoblast cells were seen in the bud stalk of control lungs, while they were restricted in a more proximal region in *Ext1*^*f/f*^*; Shh*^*cre*^ lungs (arrow head in E and F). (G-N) Immunofluorescent staining for epithelial differentiation markers for Type I pneumocytes (T1α), Type II pneumocytes (SFTPC), Club cells (SCGB1A1), Ciliated cells (Acetylated-Tubulin, Ac-Tub), Mucus cells (MUC5AC) and basal cells (P63/K5) in control lungs and *Ext1*^*f/f*^*; Shh*^*cre*^ lungs at E18.5. The epithelial differentiation was largely normal in *Ext1*^*f/f*^*; Shh*^*cre*^ lungs. Scale bars: A and B, 200μm;C and D, 100μm; E andF, 200μm; G-N, 50μm.

We next tested whether epithelial HS regulates lung patterning and cell differentiation. Although functional epithelial cells have not differentiated during branching morphogenesis, those progenitor cells can be classified into SOX2+ and SOX9+ cells that specify the proximal and distal lineages, respectively. To examine whether epithelial HS regulates lung patterning, E12.5 lungs were analyzed by the SOX2/SOX9 immunostaining. Our results showed no apparent changes in proximal-distal patterning as indicated by the SOX2 and the SOX9 marked region despite the severe defects of branching in mutant lungs (Fig 3A and 3B). Notably, SOX9+ mesenchymal cells that develop into future cartilage around the trachea appeared to be reduced (Fig 3A and 3B, inserts). In E14.5 control lungs, SOX9+ cells formed regular arranged nodes along the trachea, whereas the SOX9+ cells around the trachea in mutant lung seemed to be more dispersed and the nodes were irregularly arranged (Fig 3C and 3D, arrows), which coincides with the malformation of cartilage ring observed at E18.5 (Fig 1M and 1P). Alpha smooth muscle actin (α-SMA) staining, representing airway smooth muscle differentiation, was lining the whole developing airways and extended to the stalk of the growing lung buds in control lungs, however, in mutant lungs, its expression was restricted to a more proximal region (Fig 3E and 3F). Inactivation of epithelial *Ext1* did not affect the development of vasculature as revealed by PECAM staining (S2A–S2D Fig). We also detected a significant decline in the numbers of PH3+ cells both in the epithelium and mesenchyme of mutant lungs (S3A, S3B and S3G Fig), suggesting a reduced proliferation activity. No detectable change of apoptosis was observed (S3C–S3F Fig). During the saccular stage of lung development, the epithelial cells differentiate into multiple lineages [25]. To test whether ablation of HS in epithelium could have an impact on epithelial cell differentiation, we detected markers of major cell types of lung epithelium at E18.5. Both the type I and type II alveolar cells were presented in the cyst-like structure of the mutant lungs (Fig 3G and 3H). The club cells and ciliated cells were also presented in the cyst-like structures (Fig 3I and 3J). Basal cells and mucus cells in the trachea seem to be normally differentiated (Fig 3K–3N). These results suggest that HS in epithelium is not required for its proximal-distal patterning and differentiation but exerts a cell non-autonomous regulatory function for some aspects of the mesenchymal development.

### Epithelial HS controls SHH-FGF10 signaling

**Fig 4:**
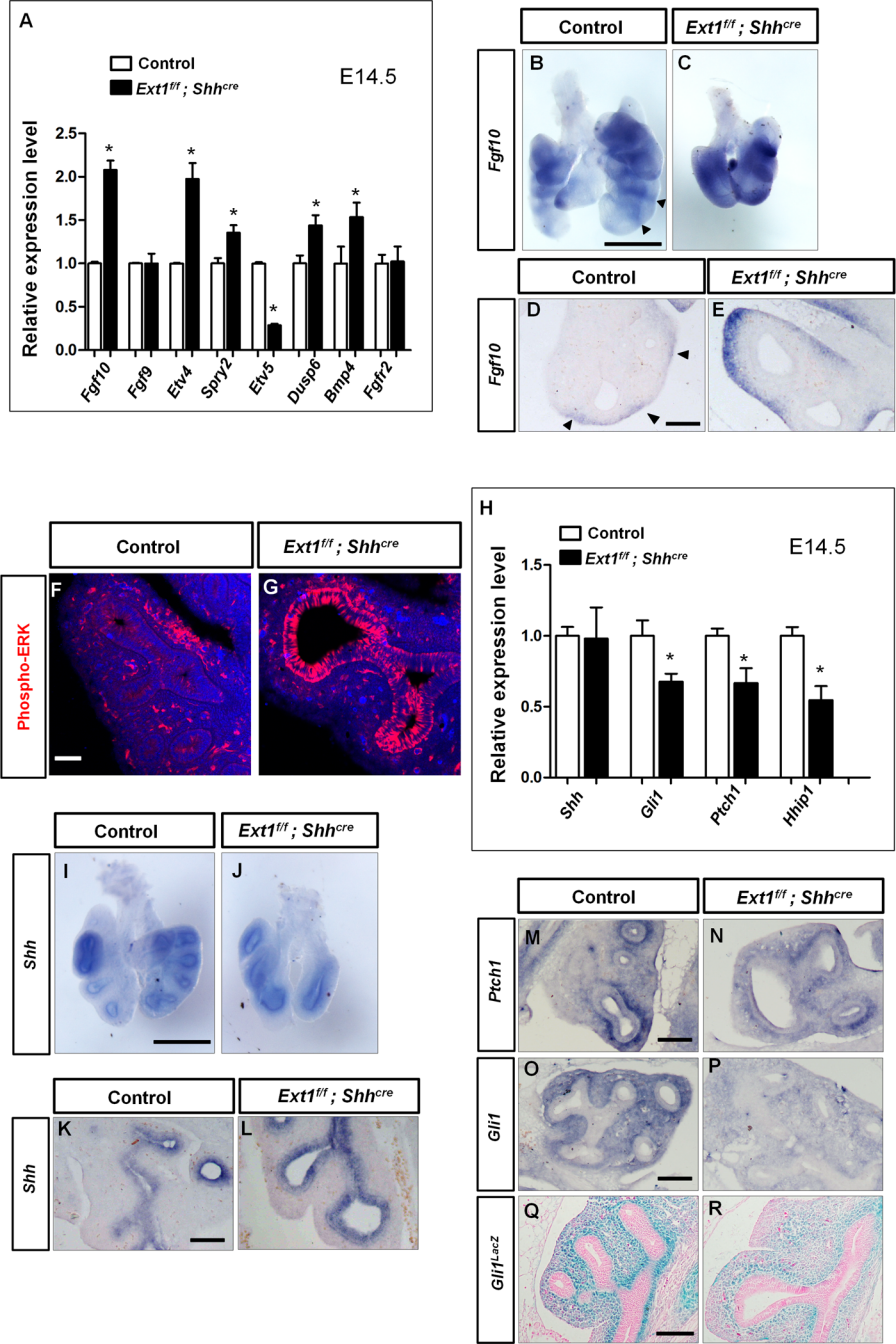
*Ext1* mutant lungs exhibit a decrease in SHH-FGF10 signaling. (A) qPCR analysis of key components of FGF signaling pathway in lungs from E14.5. The expression of *Fgf10*, *Etv4*, *Spry2*, *Dusp6* and *Bmp4* were increased, while the expression of *Etv5* was decreased in *Ext1*^*f/f*^*; Shh*^*cre*^ mutant lungs compared to control lungs. Data were presented as mean ± SEM. *p<0.05, n≥3. (B-E) Wholemount and section RNA ISH of *Fgf10* in E12.5 lungs. The *Fgf10* expression was increased in *Ext1*^*f/f*^*; Shh*^*cre*^ lungs, and its expression domain was expanded instead of the split expression pattern seen in control lungs (arrowheads in B and D). (F and G) Immunofluorescent staining for phosphorylated -ERK in E12.5 lungs. The expression domain was expanded and the intensity was much stronger in *Ext1*^*f/f*^*; Shh*^*cre*^ mutant lungs compared to control lungs, reflecting an increased FGF signaling activity. (H) qPCR analysis of key components of SHH signaling pathway in lungs from E14.5. The expression of *Shh* was not significantly changed while the SHH targets (*Gli1*, *Ptch1* and *Hhip1*) were all decreased. Data were presented as mean ± SEM. *p<0.05, n≥3. (I-L) Wholemount and section RNA ISH of *Shh* in E12.5 lungs. The expression of *Shh* was not significantly altered in *Ext1*^*f/f*^*; Shh*^*cre*^ mutant lungs. (M-P) Section RNA ISH of *Ptch1* and *Gli1* in E12.5 lungs. *Ptch1* and *Gli1* expression levels were significantly reduced. (Q-R) β-gal staining showing the expression of *Gli1*^*lacz*^ allele in control lungs (*Ext1*^*f/w*^*; Shh*^*cre*^*; Gli1*^*lacz/+*^) and mutant lungs (*Ext1*^*f/f*^*; Shh*^*cre*^*; Gli1*^*lacz/+*^). The staining intensity was decreased in mutant lungs, indicating a decreased *Gli1* expression. Scale bars: B, C, I and J, 500μm; D, E and K-R, 100μm; F and G, 50μm.

Previous studies have demonstrated that increased FGF signaling leads to abnormal branching and increased tip size [26,27], which shares striking resemblances with our *Ext1* mutant lungs, suggesting a possible involvement of aberrant FGF signaling in the defective sac-like lungs. Analysis of the expression of epithelial and mesenchymal housekeeping genes *Cdh1* and *Vimentin* revealed no changes in *Ext1*^*f/f*^; *Shh*^*cre*^ mutant lungs (S4 Fig), suggesting the proportion of epithelial cells versus mesenchymal cells was not significantly altered, confirming the validity of using *Gapdh* expression as an internal control. Indeed, qPCR revealed a 2-fold increase of *Fgf10* expression in mutant lungs compared to controls (Fig 4A). Concurrently, FGF10 targets, *Spry2*, *Dusp6*, *Etv4* and *Bmp4*, were upregulated (Fig 4A). *Etv5*, another target of FGF signaling, was decreased (Fig 4A), similar to what was observed in the *Yy1* mutant lungs [27], suggesting that epithelial HS may have pleiotropic effects on other signaling pathways. Particularly, whole mount and section RNA ISH revealed that *Fgf10* expression was uniformly high in the surrounding mesenchyme of mutant lungs, distinct from the split localization between branching tubes in control lungs (Fig 4B–4E). Expression of phosphorylated-ERK (pERK), a well-established indicator of FGF signaling activity in lungs [28,29], exhibited an expanded pattern in the dilated epithelium with much stronger fluorescent intensity compared to controls(Fig 4F and 4G),consistent with the expanded pattern of *Fgf10*. The expression of *Fgf9* and *Fgfrb2* were unchanged (Fig 4A).

Since we conditionally deleted *Ext1* in the epithelium, the cell non-autonomous effect on *Fgf10* suggests that epithelial loss of HS may have affected factors that are produced by epithelium to regulate *Fgf10* expression in mesenchyme. SHH ligand secreted from lung epithelium is essential to direct the mesenchymal development. Previous studies showed that loss of *Shh* not only resulted in branching defects, but also caused defects of trachea cartilage and airway myofibroblast [30,31]. All of these defects were observed in our *Ext1* mutant lungs. Notably, *Fgf10* is reported to be negatively regulated by SHH signaling [3]. Our data indicate that HS might regulate a hierarchical signaling pathway by modulating SHH signaling activity. However, no significant change in the expression level of *Shh* was detected by qPCR(Fig 4H). ISH analysis confirmed the qPCR results and showed no apparent changes in its expression level and pattern (Fig 4I–4L). In contrast, *Gli1*, *Ptch1* and *Hhip1* were all reduced as shown by qPCR and/or ISH experiments (Fig 4H and 4M–4P), reflecting a decreased SHH signaling activity. We also introduced a *Gli1*^*lacZ*^ reporter allele into the *Ext1* mutant. β-galactosidase (β-gal) staining revealed a decreased *Gli1* expression in mutant mesenchyme, confirming a reduction in SHH signaling activity (Fig 4Q and 4R).

FGF9 is also expressed in the developing lung epithelium, and epithelial specific overexpression of *Fgf9* causes dilation of airways and ectopic *Fgf10* expression[32]. Moreover, it was demonstrated that FGF diffusion is restricted by GAGs in source cells [15]. The unchanged *Fgf9* expression did not exclude the possibility of FGF9 over-release into the mesenchyme, which may potentially contributes to the upregulated and expanded *Fgf10* expression. However, FGF9 distribution appears to be normal in *Ext1*^*f/f*^; *Shh*^*cre*^ mutant lungs as revealed by immunofluorescent staining (S5A–S5D Fig), and consistently, the mesenchymal expression of pERK was not significantly altered in *Ext1*^*f/f*^; *Shh*^*cre*^ mutant lungs(Fig 4F and 4G). These results indicate that *Fgf9* mediated signaling pathway is not responsible for this phenotype.

Together, these data uncovered a critical regulatory role of epithelial HS in directing epithelial-mesenchymal interaction during lung branching by proper control of SHH-FGF10 signaling.

### Activation of SHH signaling partially rescues the branching phenotype in mutant lungs

**Fig 5:**
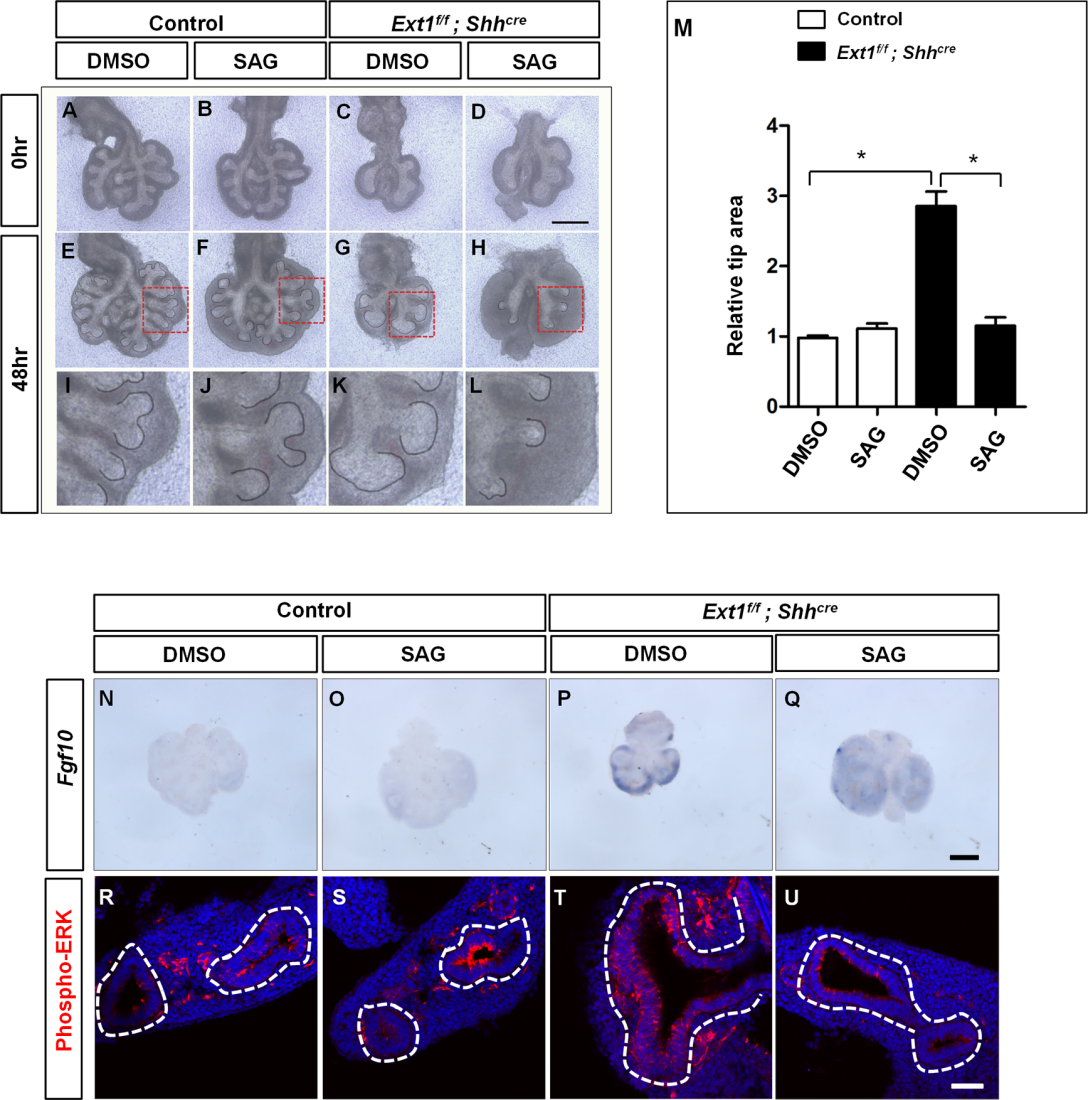
Activation of SHH attenuates the increase of tip area and FGF signaling in *Ext1* mutants. (A-H) Representative images of explant cultures treated with DMSO or SAG for 48h. Treatment of *Ext1*^*f/f*^*; Shh*^*cre*^ mutant lungs with SHH signaling agonist SAG rescued the tip dilation phenotype. The epithelium was outlined with grey line (E-H). (I -L) The corresponding enlarged view of the boxed area in (E-H). (M) Quantification of average tip area of the buds. Data were presented as mean ± SEM. *p<0.05, n = 3 for each group.(N-Q) Representative images of ISH showing *Fgf10* expression of lung explant cultures treated with DMSO or SAG for 48h. Addition of SAG attenuated *Fgf10* expression in *Ext1*^*f/f*^*; Shh*^*cre*^ mutant lungs. (R-U) Representative images of pERK staining of lung explant cultures treated with DMSO or SAG for 48h, SAG treatment attenuated epithelial pERK staining in *Ext1*^*f/f*^*; Shh*^*cre*^ mutant lungs, the epithelium was highlighted by white dashed lines. Scale bars: A-H, 500μm; N-Q,500μm; R-U, 50μm.

To test if the decreased SHH signaling activity in mutant lungs contributes to the branching defects, we manipulated SHH signaling in an explant culture system by using Smoothened agonist (SAG). We chose a final concentration of 10nM, which was close to the dose used in a previously published paper [28], *Ptch1* ISH confirmed that administration of SAG at this concentration could efficiently increase SHH signaling during the 48h of culture (S6A and S6B Fig). Control lungs treated with SAG did not exhibit significant alternations in branching number (S6C Fig) and epithelial lumen size as previously reported (Fig 5A, 5B, 5E, 5F, 5I, 5J and 5M). The mutant lungs cultured *in vitro* have the similar branching defects as what we observed *in vivo*, resulting in significantly dilated branching tips after culturing for 48hrs (Fig 5C,5G and 5K). However, SAG treatment efficiently restricted the tip size of the mutant lungs to a level that was comparable to the control lungs, although the loss of branching number was not rescued (Fig 5C, 5D, 5G, 5H, 5K, 5L and 5M). We also measured *Fgf10* expression in the cultured lungs, data from ISH analysis confirmed an attenuation of *Fgf10* expression in *Ext1*^f/f^; *Shh*^*cre*^ explants after SAG treatment (Fig 5N–5Q). Consistently, the epithelial pERK level in the branching tips of SAG treated *Ext1*^f/f^; *Shh*^*cre*^ lungs was decreased, suggesting an attenuation of FGF signaling (Fig 5R–5U). These data argue that epithelial HS is required to maintain the normal epithelium lumen size and FGF signaling, at least partly through maintaining SHH signaling activity.

The lack of rescue of branching number by SAG treatment suggested that epithelial inactivation of *Ext1* may have pleiotropic effects on other signaling pathways that affect lung branching. Previous studies have shown that WNT signaling, both canonical and non-canonical, mediates the effects of multiple factors on lung branching[33–39], and given the critical regulatory function of HS on WNT/β-catenin signaling in gut[40], we then tested whether WNT signaling is involved in our model. QPCR and ISH studies suggest that *Wnt2* expression was not significantly changed, while the expression of *Wnt7b* was reduced(S7A–S7E Fig) in *Ext1*^*f/f*^; *Shh*^*cre*^ lungs. Importantly, *Axin2*, a target for canonical WNT signaling, was significantly downregulated (S7A, S7F and S7G Fig). Further analysis reveal no changes in expression of *Wnt5a* and PCP factors implicated in non-canonical WNT signaling(S7H Fig), as well as the level of phosphorylated JNK(S7I Fig), an indicator of non-canonical WNT signaling[41].Epithelial F-actin distribution, which is also under the control of WNT/PCP pathway, was comparable between control and mutant lungs(S7J and S7K Fig). These results indicate that non-canonical WNT signaling was overall normal in *Ext1*^*f/f*^; *Shh*^*cre*^ lungs. To test whether the decreased canonical WNT signaling accounted for some of the branching phenotype, we treated lung explants with LiCl at 10mM, a previously used dose that could efficiently activate canonical WNT signaling in explant culture system [37].However, LiCl treatment was insufficient to rescue the branching defects of mutant lung explants(S7L–S7S Fig). These findings indicate that epithelial HS directly or indirectly modulates canonical WNT signaling in developing lung, however, the reduction of canonical WNT signaling may only play a minor role in branching or synergize with other dysregulated signaling pathways to contribute to some aspects of the developmental defects of *Ext1* mutant lungs.

### Epithelial HS is required for the production of biologically active form of SHH in the developing lung

**Fig 6:**
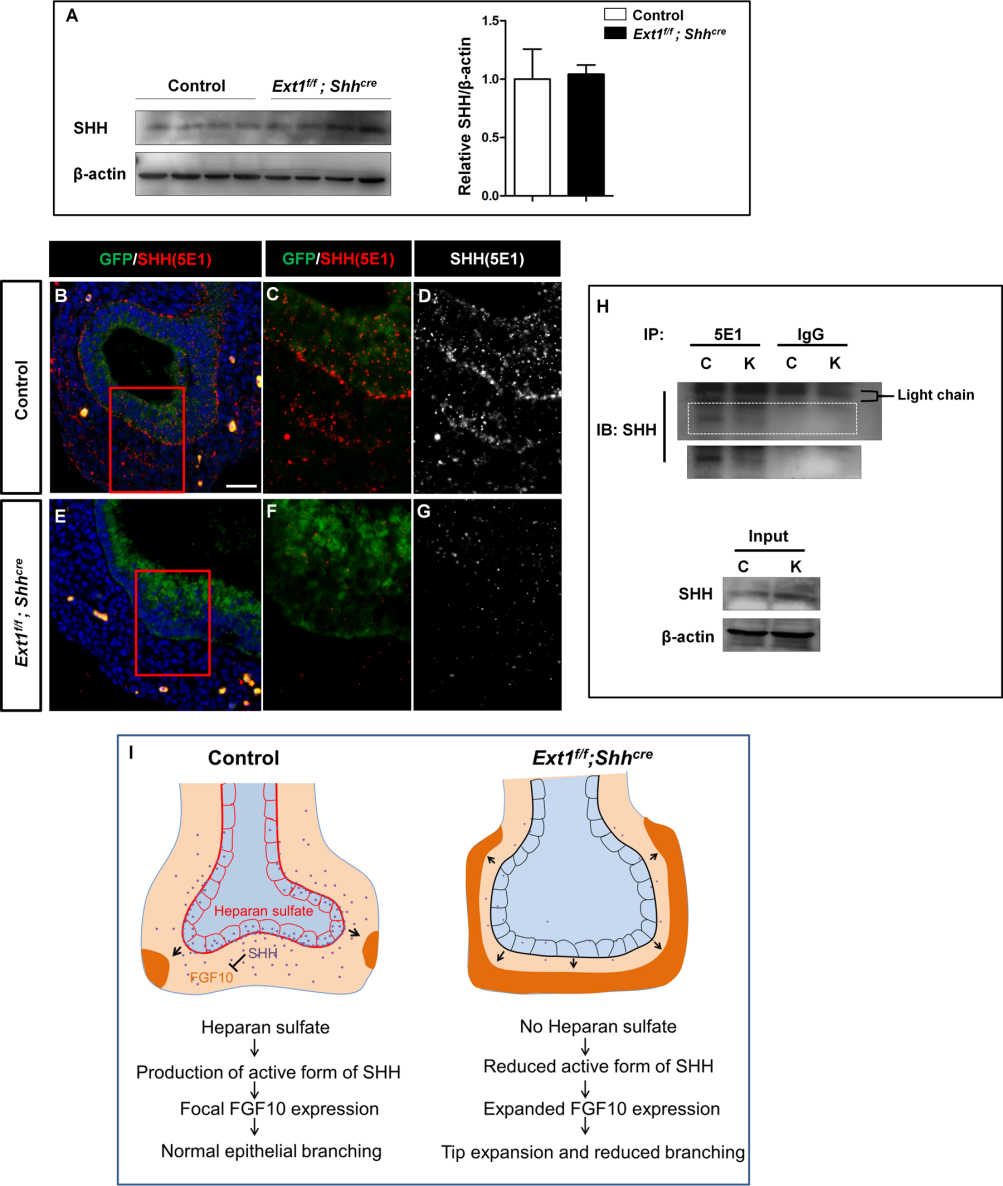
Epithelial-derived HS is required for the production of biologically active form of SHH in the developing lung. (A) Representative image of western blot analysis of SHH protein in E14.5 lungs. The protein level of SHH was not changed in *Ext1*^*f/f*^*; Shh*^*cre*^ mutant lungs compared to control lungs. (B-G) Immunoflouorescent staining for SHH(5E1) and GFP in E12.5 lungs. The GFP expression is consistent with the *Shh* mRNA. The *Ext1*^*f/f*^*; Shh*^*cre*^ mutant lungs presented less SHH particles in both the epithelium and mesenchyme. SHH particles were prone to localize on the basal side of the epithelium in control lungs, whereas the basal-localization of SHH was reduced in *Ext1*^*f/f*^*; Shh*^*cre*^ mutant lungs.(H) Immunoprecipitation(IP) of E14.5 lung homogenates using 5E1 monoclonal antibody revealed reduced 5E1-immunoreactive SHH was produced in *Ext1* mutant lungs compared with control lungs. The longer exposure of the 5E1 IP band was shown below the boxed area. C, Control; K, *Ext1* mutant. (I) Model for the regulatory role of epithelial HS in lung branching morphogenesis. During normal lung branching, SHH produced by epithelium signals to mesenchyme to ensure a focal *Fgf10* expression pattern, epithelial HS is required for the production of biologically active form (5E1 immunoreactive) of SHH ligand, possibly by controlling higher order processing. Loss of epithelial HS leads to a reduced production of biologically active form of SHH, thus causing a reduced SHH signaling and an elevated and expanded *Fgf10* expression. Decreased SHH signaling and unrestricted FGF10 signaling lead to dilated branching tips and decreased branching number. Scale bar: B and E, 20μm.

Hh-HS interactions have been extensively studied *in vivo* in *Drosophila*. It has been proposed that HSPGs in both the receiving cell and producing cell is required for Hh signaling [42–44], we asked whether HS plays conserved roles to orchestrate SHH signaling during lung development in vertebrates. First, we examined the denatured protein extracts from E14.5 lungs by western blot using a polyclonal antibody that recognizes SHH N-terminus. As shown in Fig 6A, no detectable change in SHH protein was observed, suggesting that protein stability was not affected in mutant lungs. It was previously shown that SHH shedding regulated by ECM factors such as a disintegrin and metalloprotease (ADAM) directs processing of the N terminal lipidpeptide, which exposes the zinc coordination sites (5E1 epitope) to reach the full inductive potential of the ligand[45]. And *in vitro* studies suggest that cell surface HS in Hh-producing cells spatiotemporally participates in this biological process [42,46,47]. To test whether HS is required for the spatial distribution of SHH protein in lung epithelium and mesenchyme, we stained lung sections using the monoclonal 5E1 SHH antibody. To mark the epithelium, co-staining with a GFP antibody was performed, as a *Gfp* reporter gene was also inserted into the *Shh* locus along with *Cre* in the *Shh*^*cre*^ allele, the GFP signaling is therefore consistent with *Shh* mRNA [48]. We observed that SHH 5E1 signal presented as large particles both in the epithelium and the surrounding mesenchyme in control lungs; although SHH 5E1 signals could be found in the mesenchyme, both the intensity and the number of SHH particles were prominently reduced in mutant lungs (Fig 6B–6G). We then obtained *Shh* null lungs by crossing the *Shh*^*cre/+*^ mice to generate *Shh*^*cre/cre*^ embryos. There was no detectable 5E1 staining in *Shh*^*cre/cre*^ lungs(S8A–S8D Fig), confirming the specificity of these cluster signaling. Because the 5E1 antibody recognizes the zinc coordinate site required for its receptor binding. [49], in mutant lungs, less 5E1 reactive SHH puncta reflected a low level of functional SHH protein clusters. Intriguingly, the apical-basal distribution of SHH particles in epithelium was also severely disturbed after epithelial ablation of HS: SHH were prone to be enriched in the basal side on the epithelium-mesenchyme interface (Fig 6B and 6C), where HS 3G10 signaling is most strongly presented (Fig 2A and 2C); whereas the basal distribution was not observed in mutant lungs (Fig 6E and 6F). Finally, we performed an immunoprecipitation (IP) assay with the E14.5 lung homogenates. Consistent with the above data, we found that 5E1 immunoprecipitated SHH protein was significantly reduced (Fig 6H), which confirms the reduction of 5E1 immunoreactive SHH protein. These results suggested a possibility that the HS chains in the epithelial cell surface and basement membrane possibly serve as an important SHH repository network for its processing and later transportation, assisting to maintain a proper concentration of biologically active SHH in the surrounding mesenchyme.

## Discussion

Although it is well understood that multiple morphogens regulate interconnected transcriptional network to control lung development, how the cell surface and ECM molecules are involved in these morphogenic processes is still incompletely understood. HS has been reported to regulate branching morphogenesis of multiple organs such as mammary gland, submandibular gland, and lacrimal gland [16–20,50–52]. In a number of contexts, loss of HS leads to disorders of FGF signal transduction. Thus, we had initially speculated that loss of HS in epithelial cells could lead to an FGF loss of function phenotype during lung development. To our surprise, an FGF gain of function phenotype was observed. We demonstrate that lung epithelial derived HS, presented both on the cell surface and basement membrane ECM, is critical for the maintenance of SHH signaling activity, thereby exerting an indispensable role in branching morphogenesis. Ablation of HS synthetase *Ext1* in the epithelium resulted in strikingly reduced levels of biologically active (5E1-immunoreactive) SHH particles both on the epithelium and mesenchyme and subsequently reduced signaling activity in the receiving cells, causing an unrestricted *Fgf10* expression, which ultimately led to expanded branching tips and reduced branching number (Fig 6I). Our study highlights the critical functions of HS during lung development.

In *Ext1* conditional knockout lungs, we observed an apparent decline of cell proliferation of the epithelial and mesenchymal cells; in addition, epithelial differentiation was largely unaffected, whereas the airway myofibroblast and cartilage development were severely disrupted, suggesting there is a cell non-autonomous requirement of the epithelial HS for multiple aspects of the lung development. Lung development is critically regulated by interactions of signaling molecules between epithelium and mesenchyme, among which SHH is one of the most studied molecules. SHH, produced by epithelium cells, signals to the adjacent mesenchyme cells to support mesenchyme cell development and regulate the expression of growth factors such as *Fgf10* and *Wnt2*[31]. FGF10 in turn signals to the epithelium, which is critically required for domain branching, bifurcation and epithelium extension [1]. *Shh* null mutant failed to form a functional lung, as a result of branching halt [31]. Recent studies showed conditional knockout of transcriptional factors, such as ETV and YY1 in lung epithelium reduced *Shh* expression, which subsequently broke the balance between SHH and FGF signaling, resulted in enlarged tips and decreased branching number [27,28]. These studies suggested that a fine-tuning of SHH-FGF signaling is essential for lung branching morphogenesis. Our data showed that epithelial deletion of HS caused similar branching defects as described above, including enlarged tip bud size, decreased branching number, and expanded *Fgf10* expression. We further provided evidence that SHH signaling was reduced in mutant lungs, highlighting an essential requirement for HS in dictating the SHH-FGF10 signaling cascade.

Prior to our study, several studies have examined the function of HS in lung morphogenesis. The O-sulfated HS in the lung epithelium was proven to be required for FGF10-FGFR2b binding and signaling in an organ culture system [22,23]. In support of this notion, genetic ablation of *Hs6st* in lacrimal gland disrupted FGF10-FGFR2b signaling [16]. However, mice lacking *Hs6st* seemingly did not bear lung branching defects [24]. In *Ext1* mutant lungs, we observed an increased FGF signaling, as exemplified by increased expression of multiple FGF targets and pERK level, which stands in contrast to the previous notion that HS is required for FGF signaling transduction, suggesting that the overabundance of FGF10 ligand in the local environment may compensate the decreased FGF10-FGFR2b interaction, or an HS-independent mechanism that facilitates FGF10-FGFR2b binding may exist in the lung epithelium when HS is removed.

Our data indicate that the branching defects in *Ext1* mutant lungs mimicked those with increased FGF signaling [26–28,53], this is likely resulted from an increased and expanded *Fgf10* expression in the mesenchyme. Thus, we speculated that signaling that acts upstream of FGF10 might be altered. Indeed, we have detected a retard of SHH signaling in *Ext1* mutants, without obvious changes in its mRNA level and protein stability, indicating that HS-SHH interaction in the producing cell maintains the signaling activity possibly by a higher order processing of SHH. The current knowledge on HS-Hh interaction is mostly obtained from *in vitro* experiments and *in vivo* experiments on lower organisms. In *ttv* (*Ext1* in *Drosophila*) null *Drosophila* embryo, Hh failed to move to its target cells [54]. Hh also failed to move through Glypican-mutant cells[44]. Biochemical studies suggested that the multimerization of vertebrate SHH induces long range signaling activity [55,56], and SHH multimer formation and release is dependent on HS[42,45,46]. In particular, it was previously shown that nanoscale oligomerized Hh interacts with HSPGs to form the densely packed multimers, which is essential to present itself on cell surface or in lipoproteins for long range signaling [43]. Recent evidence indicates that HS in the heteroprotein hubs can attract sheddases and sheddase activators such as ADAM to help SHH multimers to undergo N-terminal processing, a procedure required to reveal its zinc coordinate sites and results in its functional activation.[42,45–47]. In support of this model, after abrogating HS synthesis in lung epithelium, we observed a dramatic reduction of the 5E1-immunoreactive SHH particles both in the epithelium and mesenchyme, with a reduction of SHH signaling activity. We speculate that abrogation of epithelial HS changed ECM dynamics required for SHH processing, this notion is supported by a recent finding showing compromised SHH signaling in the umbilical cord of ADAMTS9 mutant mice[57]. Observations in *Ext1* null embryos found that less IHH(5E1) protein was associated with endodermal cell surface although its protein level was not changed, similar to our finding [14]. In this study, we observed strong 3G10 epitope of HS in the basement membrane (Fig 2A and 2C). Previous biochemical analysis demonstrated that a higher sulfation level of HS chains leads to a higher affinity to SHH protein [58]. This could possibly explain why we observed a strong signal of SHH on the basal side of the epithelium, because the embryonic lung basement membrane HS are highly O-sulfated rather than N-sulfated [23]. In the context of developing ventral spinal cord, Sulf1, an enzyme that reduces the 6-O-sulfation of HS and is expressed in SHH producing cells, was required for the production of biologically active form of SHH [59–61], demonstrating that SHH signaling is precisely regulated by the dynamic change of HS sulfation pattern. Additional genetic evidence is required to understand the role of HSPGs and HS modification enzymes in SHH signaling more conclusively in the developing lung. Moreover, it would also be interesting to investigate the potential role of mesenchymal HS during lung development.

Our results support that epithelial HS is required for the production of fully activated SHH ligand to ensure normal lung branching. Although the tip dilation phenotype and hyperactivation of epithelial FGF signaling in mutant lungs can be attenuated by SAG, the lack of rescue of branching number suggests that there must be additional signaling pathways that act coordinately with SHH-FGF10 signaling to contribute to the phenotype. Alternation of multiple signalings have been reported to have similar defects in *Ext1* mutant lungs. For example, *Fgf9* overexpression in lung epithelium induces upregulation of *Fgf10*, and causes epithelium expansion[32]; inactivation of *Frizzled 2* results in dilated branching tips through modulation of non-canonical WNT signaling[35]. However, we did not find evidence of changes in FGF9 signaling or non-canonical WNT signaling. Intriguingly, *Ext1* mutant lungs exhibited a dramatically decreased *Wnt7b* expression and canonical WNT signaling. Despite the failure of LiCl to rescue the branching, the decreased canonical WNT signaling may work synergistically with other dysregulated pathways to affect lung morphogenesis in *Ext1* mutant lungs, further genetic study is required to analyze this issue. In addition to the disruption of external cues required for lung branching, loss of HS may potentially result in changes of ECM composition, which may cause intrinsic cellular defects that affect lung branching. Proper deposition of Collagen and Integrin is required for the migration activity of lung epithelial[62–64], and a recent finding in *C*. *elegan* provide evidence of the requirement of HS for neural cell migration[65]. Whether HS interacts with these factors to contribute to the lung branching phenotype needs to be further explored.

In conclusion, our study demonstrates epithelial derived HS is critical in maintaining SHH signaling activity, thus dictates the epithelial-mesenchymal interaction during lung development. As Hh signaling is proposed to be involved in lung homeostasis and tumorigenesis [66,67], it is possible that HS is involved in the pathogenesis of many lung diseases such as cancer, fibrosis and COPD, in addition to its essential role during organogenesis.

## Materials and methods

### Ethics statement

Animal experiments were performed following the Institutional guidelines and approved by the Institutional Animal Care and Use Committee of Institute of Zoology, Chinese Academy of Sciences(approve number: AEI-09-012014).

### Animals

Mouse lines for *Ext1*^*f/f*^, *Gli*^*lacZ*^, *Shh*^*cre*^ were described elsewhere[48,68,69]. All lines were maintained on a mixed background. Mice were housed in a normal 12 light/12 dark cycle in the SPF (Specific-Pathogen-Free) animal facility of Institute of Zoology, Chinese Academy of Sciences, Beijing, China. *Shh*^*cre*^ line was used to delete *Ext1* specifically in lung epithelial cells during lung morphogenesis. Mutant mice (*Ext1*^*f/f*^; *Shh*^*cre*^) were generated by crossing *Ext1*^*f/+*^*; Shh*^*cre*^ male with *Ext1*^*f/f*^ female. Embryos were harvested and analyzed from time-mated females at indicated times (noon of the next day after mating counted as 0.5 day of gestation). *Ext1*^*f/+*^*; Shh*^*cre*^ littermates were referred as controls.

### Histology and immunofluorescent staining

Embryos were harvested at indicated times and fixed in 4% paraformaldehyde at 4°C overnight, followed by dehydrated in a series of ethanol and embedded in paraffin for sectioning. For immunohistochemistry staining, deparaffinized and rehydrated paraffin sections (5μm) were microwaved in sodium citrate buffer (pH6.0). The primary antibodies were listed in S1 Table, fluorescent conjugated secondary antibodies were all from Jackson Immunoresearch. For Immunofluorescent staining of SOX2, SHH(5E1)[70], pERK, FGF9, P63,10E4 and 3G10, ABC immunoperoxidase detection systems (Vector PK-6200 Laboratories) was used to introduce HRP. The Cyanine 3 Tyramide Signal detection was performed according to the guidelines of TSA amplification system (PerkinElmer). For 3G10 staining, sections were pretreated by heparanase III (400 mU/ul, Sigma) at 37°C for 2 h to expose neo-epitope site. TUNEL staining was performed using the DeadEnd™ Fluorometric TUNEL System (Promega) according to manufacturer’s protocol. F-actin staining was performed on 12μm-cryosections using Alexafluor546 conjugated phalloidin (Invitrogen). All the slides were imaged on a Zeiss LSM 780 laser-scanning confocal microscope under the same laser exposure between control and mutant lungs.

### β-galactosidase (β-gal) staining

β-gal staining was performed as previously described [28] with little modification. Embryos and lung tissues were fixed in 4% paraformaldehyde at 4°C for 2 hours. Fixed embryos were washed repeatedly with rinse buffer (5 mM EGTA, 0.1% Deoxycholate, 0.02% NP40, 2 mM MgCl2 in PBS) for 15 mins. Samples were then stained with 1mg/ml X-gal solution (5 mM K3Fe(CN)6, 5 mM K4Fe(CN)6, 5 mM EGTA, 0.1% Deoxycholate, 0.02% NP40, 2 mM MgCl2 in PBS) overnight. After staining, tissues were rinsed with PBS, postfixed with 4% paraformaldehyde and embedded in paraffin.

### Alcian blue cartilage staining

Respiratory tracts were dissected and stained with alcian blue solution as previously described [27]. The tracheas were rinsed with PBS and immersed in 70% glycerol for image.

### RNA *in situ* hybridization (ISH)

cDNA fragments amplified from mouse lung cDNA were used as probe templates. The primers were listed in S2 Table. RNA riboprobes were produced according to the instruction of DIG RNA Labeling Kit (Roche). Sense riboprobes transcribed by T3 RNA polymerase were used as negative control. ISH on paraffin sections was performed as previously described [37]. Whole mount in situ hybridization was carried out using an optimized protocol [71].

### Organ culture

Lungs were harvested from E11.5 embryos in DMEM/F12 (Corning), and then placed onto Nucleopore Track-Etch membranes (8μm Whatman) to expand lungs at the air/medium interface. *Ex vivo* lungs were cultured in DMEM/F12 with 100U penicillin and 100μg streptomycin (Thermo Fisher Scientific) at 37°C in 5% CO2. SHH signaling activator, smoothened agonist SAG (10nM, SelleckChem) was added into culture media. DMSO was used as a vehicle control. Bright field images were captured 48 hours after SAG administration, and quantification was performed using Image J software.

### Quantitative RT-PCR (qRT-PCR)

RNA isolation with Trizol (Invitrogen) was performed according to manufacturer’s instructions. First strand cDNA was reverse transcribed using GoScript Reverse Transcription System (Promega). Quantitative PCR was performed using GoTaq qPCR Master Mix (Promega) and amplification was quantified by CFX-96 system (Bio-rad). Expression levels were normalized to *Gapdh*. At least three biological replicates are performed for each experiment. Primer sequences used are listed in S2 Table.

### Immunoprecipitation (IP) and western blot

Lungs were dissected from Embryos in PBS, and homogenized using Dounce tissue grinder in RIPA lysis buffer (Beyotime) containing protease inhibitor and phosphatase inhibitor (Roche). For each IP, approximately 80mg pooled lung tissues were homogenized in 500μl lysis buffer, lung lysates were centrifuged at 15000g for 20min,supernantants were collected. 40μl Protein A/G plus agrose beads(Santa Cruz biotech, Inc) and 5μg 5E1 antibody (DSHB) was used for each IP reaction. After rotation at 4°C overnight, the beads were washed, reconstituted and boiled in 30ul 1X SDS loading buffer. For western blot analysis of tissue lysates, approximately 80μg total protein was reduced in 4X SDS loading buffer and subjected to 12% SDS-PAGE. Immunobloting was performed on PVDF membrane. Membranes were incubated with antibodies against SHH (goat polyclonal, AF464, RD Systems Inc., 1:300),β-actin(mouse monoclonal, CW0096M, Cwbiotech, 1:2000) and p-JNK(rabbit polyclonal, 9251, Cell signaling technology,Inc., 1:1000) at 4°C overnight. HRP conjugated anti-mouse, anti-rabbit and anti-goat secondary antibodies (Jackson Immunoresearch) were used and peroxidase activity was detected by SuperSignal West Pico Chemiluminescent Substrate (Thermo Scientific). Chemiluminescent signal was detected and pictures were captured by GE ImageQuant LAS 4000. Quantification was performed using the Image J software.

### Statistical analysis

Data are shown as means ± SEM. For comparative analysis, Unpaired Student’s two-tailed t-test and graphs were achieved using GraphPad Prism6. p<0.05 was considered statistically significant.

## Supporting information

S1 Fig Lung branching phenotype, lobe specification defects and trachea-esophagus fistula in *Ext1* mutant lungs. (A and B) Whole mount view of E12.5 lungs showing the reduced branching number and enlarged branching tips in *Ext1*^*f/f*^*; Shh*^*cre*^ mutant lungs. (C and D) A small proportion of the *Ext1*^*f/f*^*; Shh*^*cre*^ mutant lungs showed isosymmetric lobe patterning, only left (L) and right (R) lungs were seen, while the cranial (Cr), medial (Md), caudal (Cd), and accessory (Ac) lobes were seen in control lungs. (E-F) The esophagus was fused to the trachea in *Ext1*^*f/f*^*; Shh*^*cre*^ mutant lungs.(G-H) Transverse section at the thymus level showing the failure of trachea-esophagus separation in *Ext1*^*f/f*^*; Shh*^*cre*^ mutant lungs, Scale bars:200μm.
(TIF)

S2 Fig Inactivation of epithelial *Ext1* does not affect the development of vasculature. (A-D) Immunofluorescent staining of vascular endothelial cell marker PECAM at E14.5 (A and B) and E18.5 (C and D) showing the vasculogenesis is normal in *Ext1*^*f/f*^*; Shh*^*cre*^ mutant lungs. Scale bar: 50μm
(TIF)

S3 Fig *Ext1* mutant lungs exhibit an overall reduction of proliferation in epithelium and mesenchyme. (A and B) Immunofluorescent staining for PH3 at E12.5 showed the decreased mitosis in the epithelium and mesenchyme of *Ext1*^*f/f*^*; Shh*^*cre*^ mutant lungs. (C- F) TUNEL staining showed no obvious change in apoptosis at E12.5 (C and D) and E18.5(E and F) between control and *Ext1*^*f/f*^*; Shh*^*cre*^ mutant lungs. (G) Quantification of the mitotic cells (PH3+) in control lungs and *Ext1*^*f/f*^*; Shh*^*cre*^ mutant lungs. *p<0.05, n = 3 for each group. Scale bars: A-F, 50μm.
(TIF)

S4 Fig Inactivation of epithelial *Ext1* does not significantly alter the proportion of epithelial cells and mesenchymal cells. QPCR analysis of the housekeeping genes for epithelium(*Cdh1*) and mesenchyme(*Vimentin*) at E14.5 revealed no changes in their expression and their ratio. *p*>0.05,n = 3.
(TIF)

S5 Fig FGF9 distributed normally in *Ext1* mutant lungs. Immunofluorescent staining of FGF9 of E12.5 lungs.FGF9 proteins were found in the epithelium and mesenchyme. No evidence of aberrant FGF9 distribution was found in *Ext1*^*f/f*^*; Shh*^*cre*^ mutant lungs. Scale bar: A and B, 20μm.
(TIF)

S6 Fig The effect of SAG treatment on SHH signaling activity and branching number. (A and B) ISH analysis of *Ptch1* expression of wild type lungs treated with DMSO or 10nM SAG for 48h in explant culture. *Ptch1* expression was significantly increased following SAG treatment.(C) The statistical analysis of branching endpoints of lungs following SAG treatment, no significant change was observed in both the control and mutant lungs. *p*>0.05,n = 3. (D-O) The underlying images used for quantification for (C) and Fig 5M.
(TIF)

S7 Fig Canonical WNT signaling activity is decreased in *Ext1* mutant lungs. (A) QPCR analysis for the expression of ligands (*Wnt2* and *Wnt7b*) and target(*Axin2*) for canonical WNT signaling at E14.5.*p<0.05 vs. Control, n≥4. (B-G) ISH analysis confirmed the reduction of *Wnt7b* and *Axin2* expression in *Ext1*^*f/f*^*; Shh*^*cre*^ mutant lungs at E12.5. (H) QPCR analysis for the expression of ligand (*Wnt5a*) and factors (*Arhgef19*,*Celsr1* and *Vangl2*) involved in non-canonical WNT signaling showed no change between control and mutant lungs at E14.5. *p*>0.05,n≥3. (I)Western blot analysis of p-JNK, an indicator of non-canonical WNT signaling, also showed no change. (J and K) Phalloidin staining of E12.5 lungs showing the F-actin distribution was comparable between control and *Ext1*^*f/f*^*; Shh*^*cre*^ mutant lungs. (L-S)Treating lung explants with the activator for canonical WNT signaling, LiCl, was unable to rescue the branching defects in *Ext1*^*f/f*^*; Shh*^*cre*^ mutant lungs. Lungs were dissected at E11.5 and cultured in the presence or absence of LiCl(10mM) for 48h. Scale bars: B-G, 100μm; J and K, 20μm; L-S, 500μm.
(TIF)

S8 Fig Validation of the specificity of SHH(5E1) fluorescent signal. (A-D) Immunofluorescent staining of *Shh*^*cre/+*^ lungs and *Shh* null lungs (*Shh*^*cre/cre*^) using 5E1 antibody. The cluster signal were found both in the epithelium and the mesenchyme of the control lungs with strong stainings in the basement membrane. *Shh* null lungs were negative for the signal. Scale bar: A and B, 20μm.
(TIF)

S1 Table Primary antibody information for IHC. (DOCX)

S2 Table Primers used in this study. (DOCX)
