# Supplementary figures and images for: Epithelial heparan sulfate regulates Sonic Hedgehog signaling in lung development
Source: PLoS Genet. 2017 Aug 31;13(8):e1006992. doi: 10.1371/journal.pgen.1006992 (PMC5597256; doi:10.1371/journal.pgen.1006992)

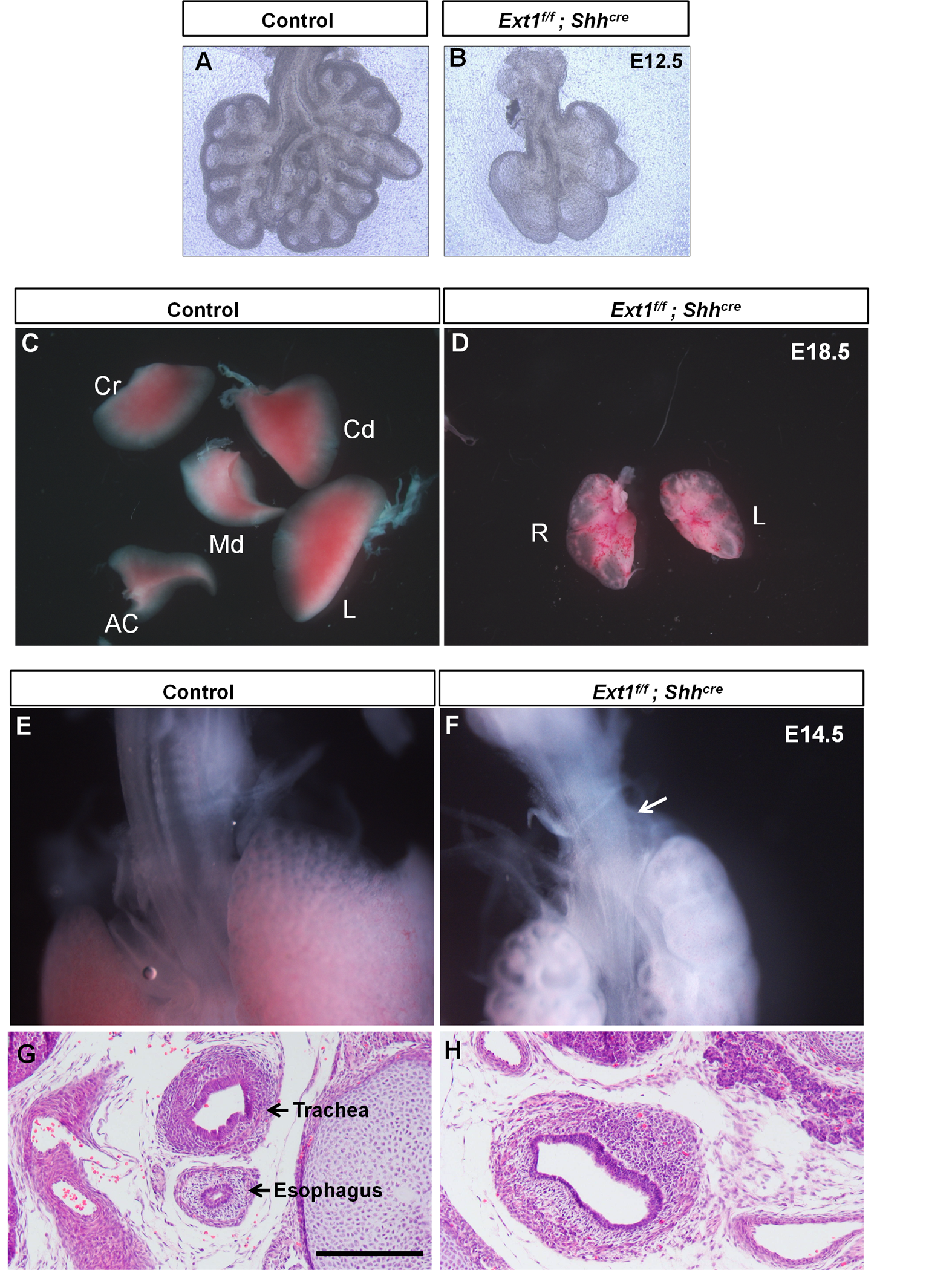

Supplement: S1 Fig — (A and B) Whole mount view of E12.5 lungs showing the reduced branching number and enlarged branching tips in Ext1f/f; Shhcre mutant lungs. (C and D) A small proportion of the Ext1f/f; Shhcre mutant lungs showed isosymmetric lobe patterning, only left (L) and right (R) lungs were seen, while the cranial (Cr), medial (Md), caudal (Cd), and accessory (Ac) lobes were seen in control lungs. (E-F) The esophagus was fused to the trachea in Ext1f/f; Shhcre mutant lungs.(G-H) Transverse section at the thymus level showing the failure of trachea-esophagus separation in Ext1f/f; Shhcre mutant lungs, Scale bars:200μm. (TIF) [file pgen.1006992.s001.tif]

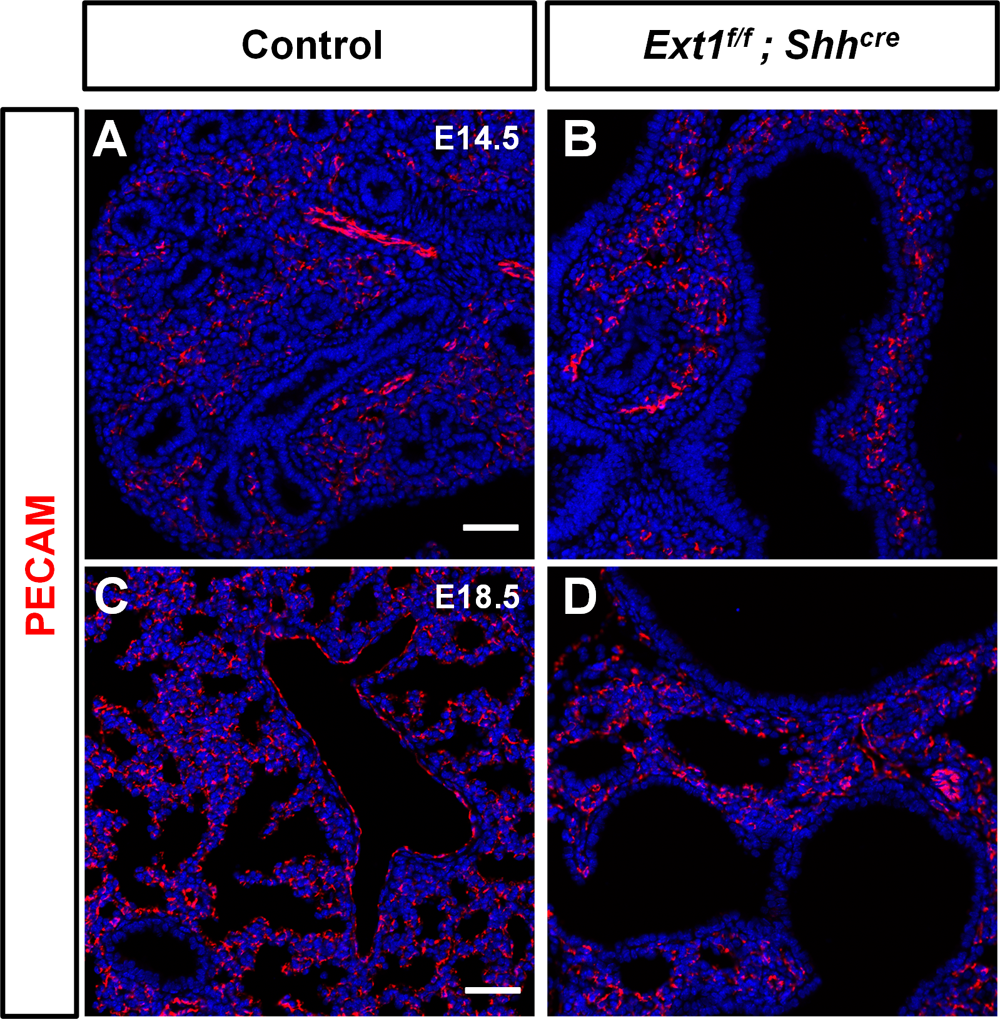

Supplement: S2 Fig — (A-D) Immunofluorescent staining of vascular endothelial cell marker PECAM at E14.5 (A and B) and E18.5 (C and D) showing the vasculogenesis is normal in Ext1f/f; Shhcre mutant lungs. Scale bar: 50μm (TIF) [file pgen.1006992.s002.tif]

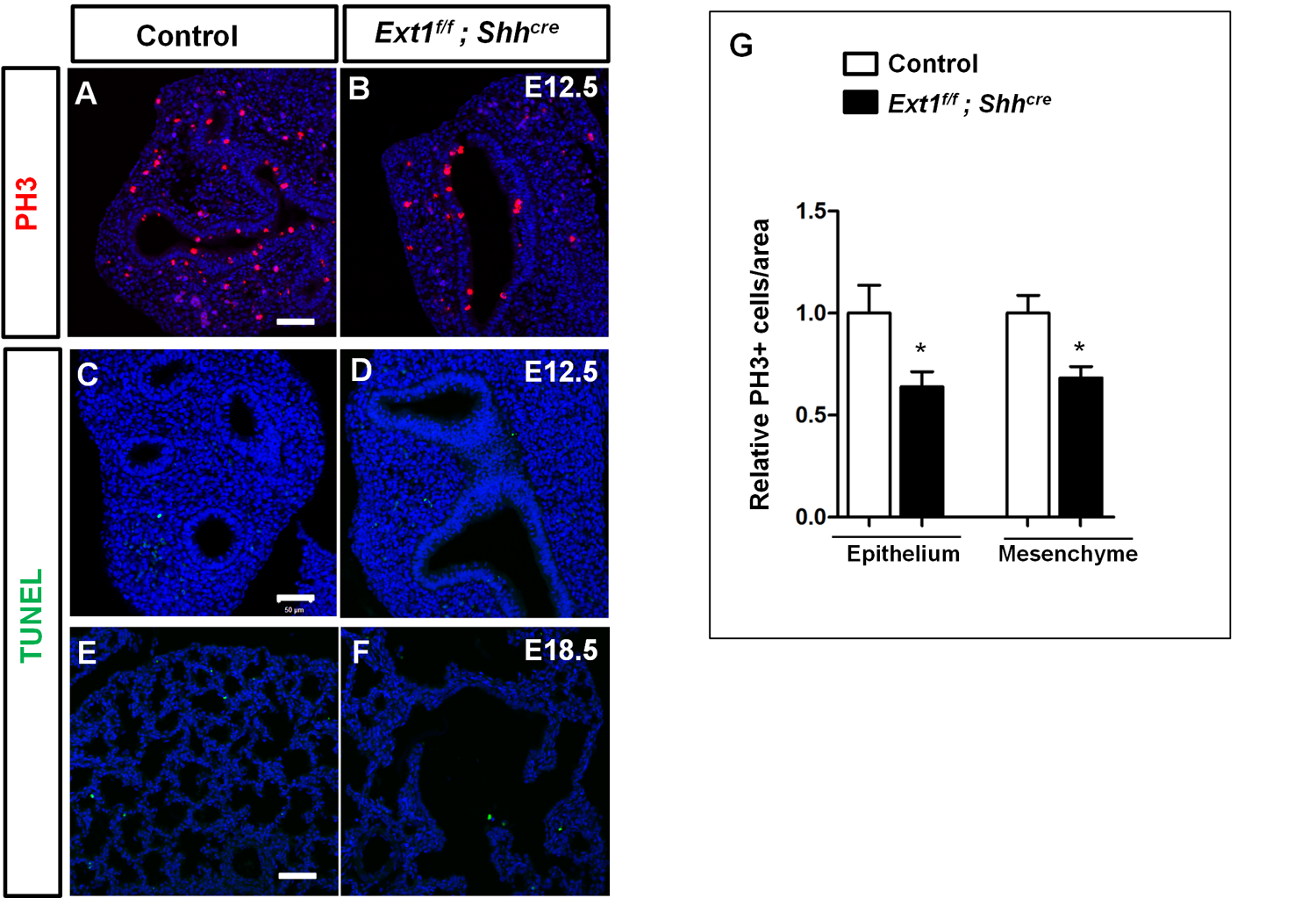

Supplement: S3 Fig — (A and B) Immunofluorescent staining for PH3 at E12.5 showed the decreased mitosis in the epithelium and mesenchyme of Ext1f/f; Shhcre mutant lungs. (C- F) TUNEL staining showed no obvious change in apoptosis at E12.5 (C and D) and E18.5(E and F) between control and Ext1f/f; Shhcre mutant lungs. (G) Quantification of the mitotic cells (PH3+) in control lungs and Ext1f/f; Shhcre mutant lungs. *p<0.05, n = 3 for each group. Scale bars: A-F, 50μm. (TIF) [file pgen.1006992.s003.tif]

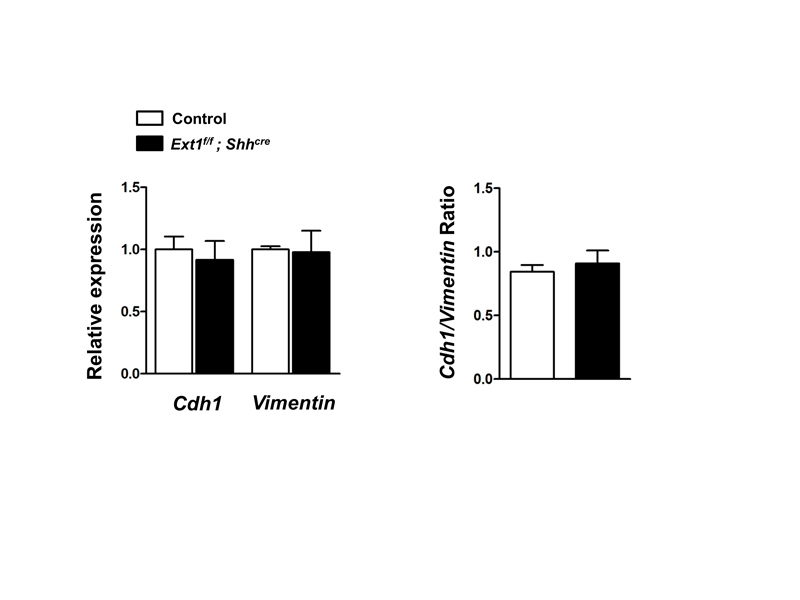

Supplement: S4 Fig — QPCR analysis of the housekeeping genes for epithelium(Cdh1) and mesenchyme(Vimentin) at E14.5 revealed no changes in their expression and their ratio. p>0.05,n = 3. (TIF) [file pgen.1006992.s004.tif]

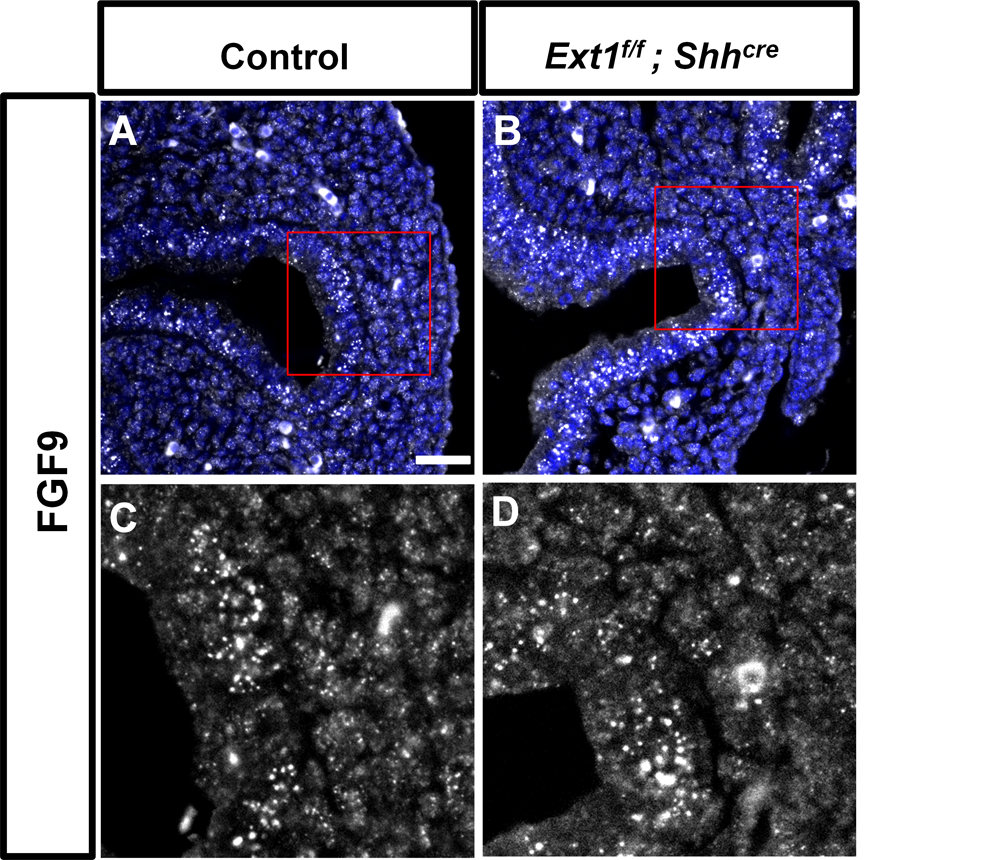

Supplement: S5 Fig — Immunofluorescent staining of FGF9 of E12.5 lungs.FGF9 proteins were found in the epithelium and mesenchyme. No evidence of aberrant FGF9 distribution was found in Ext1f/f; Shhcre mutant lungs. Scale bar: A and B, 20μm. (TIF) [file pgen.1006992.s005.tif]

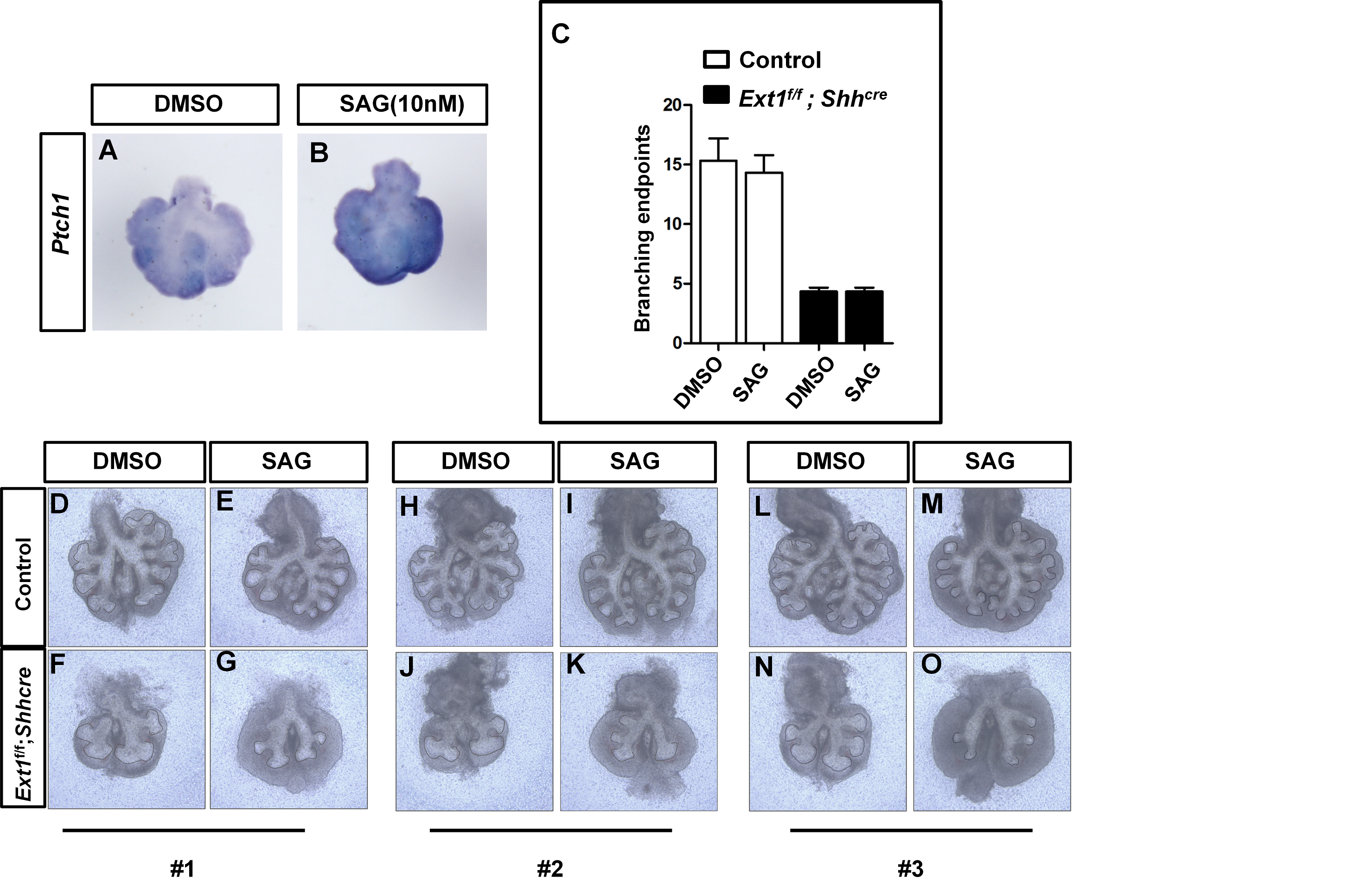

Supplement: S6 Fig — (A and B) ISH analysis of Ptch1 expression of wild type lungs treated with DMSO or 10nM SAG for 48h in explant culture. Ptch1 expression was significantly increased following SAG treatment.(C) The statistical analysis of branching endpoints of lungs following SAG treatment, no significant change was observed in both the control and mutant lungs. p>0.05,n = 3. (D-O) The underlying images used for quantification for (C) and Fig 5M. (TIF) [file pgen.1006992.s006.tif]

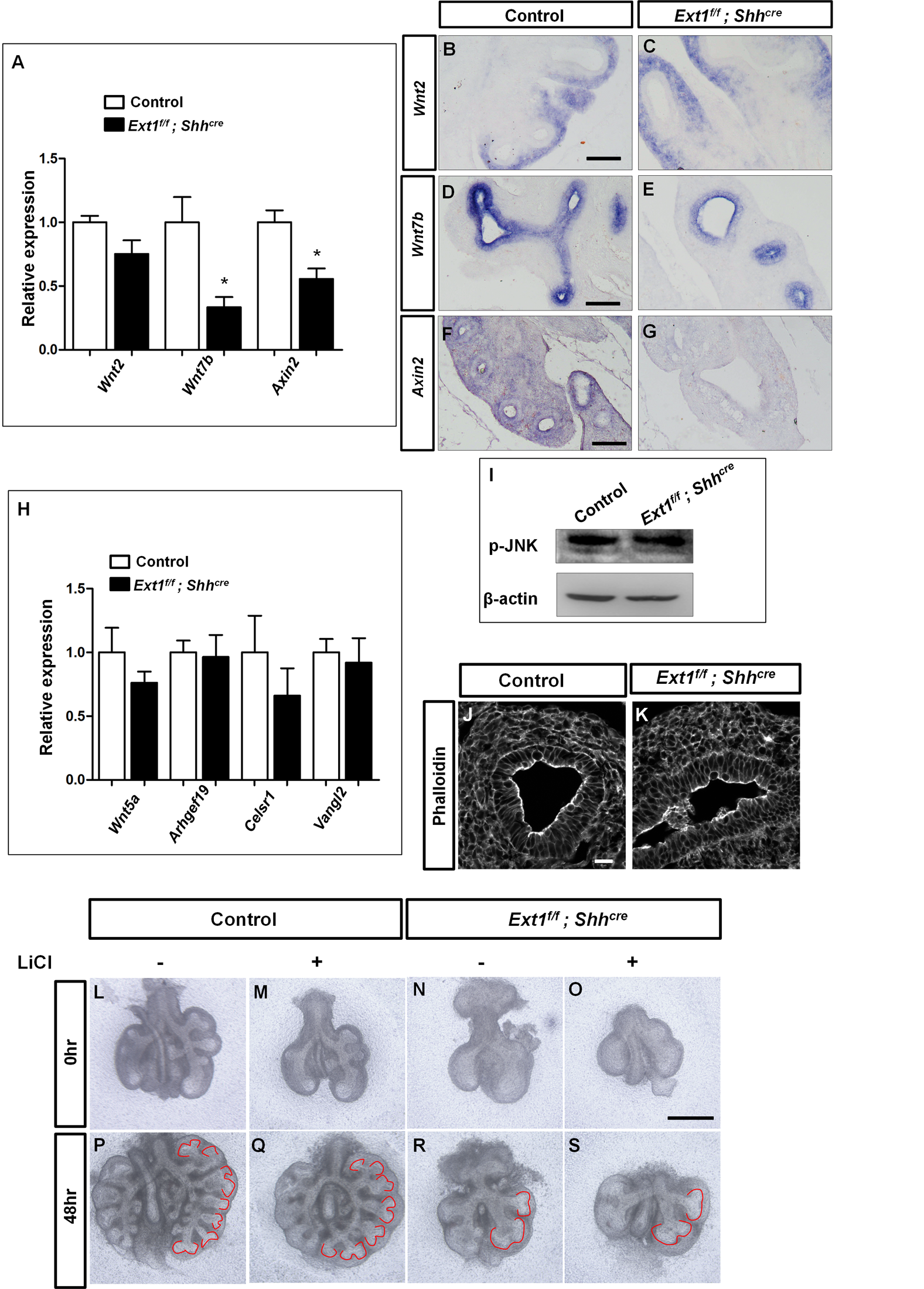

Supplement: S7 Fig — (A) QPCR analysis for the expression of ligands (Wnt2 and Wnt7b) and target(Axin2) for canonical WNT signaling at E14.5.*p<0.05 vs. Control, n≥4. (B-G) ISH analysis confirmed the reduction of Wnt7b and Axin2 expression in Ext1f/f; Shhcre mutant lungs at E12.5. (H) QPCR analysis for the expression of ligand (Wnt5a) and factors (Arhgef19,Celsr1 and Vangl2) involved in non-canonical WNT signaling showed no change between control and mutant lungs at E14.5. p>0.05,n≥3. (I)Western blot analysis of p-JNK, an indicator of non-canonical WNT signaling, also showed no change. (J and K) Phalloidin staining of E12.5 lungs showing the F-actin distribution was comparable between control and Ext1f/f; Shhcre mutant lungs. (L-S)Treating lung explants with the activator for canonical WNT signaling, LiCl, was unable to rescue the branching defects in Ext1f/f; Shhcre mutant lungs. Lungs were dissected at E11.5 and cultured in the presence or absence of LiCl(10mM) for 48h. Scale bars: B-G, 100μm; J and K, 20μm; L-S, 500μm. (TIF) [file pgen.1006992.s007.tif]

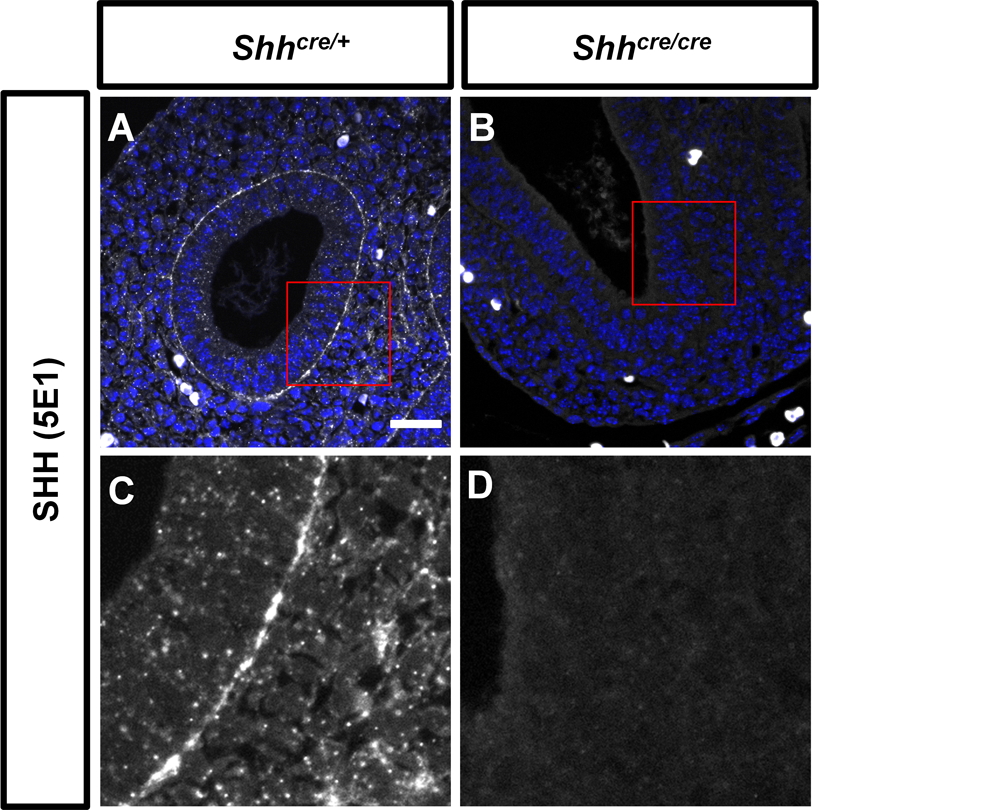

Supplement: S8 Fig — (A-D) Immunofluorescent staining of Shhcre/+ lungs and Shh null lungs (Shhcre/cre) using 5E1 antibody. The cluster signal were found both in the epithelium and the mesenchyme of the control lungs with strong stainings in the basement membrane. Shh null lungs were negative for the signal. Scale bar: A and B, 20μm. (TIF) [file pgen.1006992.s008.tif]
