# Supplementary material for: Epithelial heparan sulfate regulates Sonic Hedgehog signaling in lung development
Source: PLoS Genet. 2017 Aug 31;13(8):e1006992. doi: 10.1371/journal.pgen.1006992 (PMC5597256; doi:10.1371/journal.pgen.1006992)
Supplement: S1 Table — (DOCX) [file pgen.1006992.s009.docx]

**S1 Table**

|  | Manufacturer | Catalog | Dilution |
| --- | --- | --- | --- |
| α-SMA | Abcam | ab7817 | 1:100 |
| Acetylated Tubulin | Sigma | T7451 | 1:500 |
| FGF9 | Abcam | Ab9743 | 1:100 (TSA amplification) |
| GFP | Abcam | ab13970 | 1:400 |
| HS (3G10) | AMSBIO | 370260-S | 1:100 (TSA amplification) |
| HS (10E4) | AMSBIO | 370255-S | 1:100 (TSA amplification) |
| Keratin5 | Covance | PRB-160P-100 | 1:200 |
| MUC5AC | Abcam | ab3649 | 1:100 |
| P63 | Abcam | ab735 | 1:50(TSA amplification) |
| PECAM | Abcam | ab28364 | 1:100 |
| Perk | Cell Signaling Technology | 4370 | 1:200 (TSA amplification) |
| PH3 | Cell Signaling Technology | 9701 | 1:200 |
| SFTPC | Millipore | ABC99 | 1:300 |
| SCGB1A1 | Santa Cruz | sc-9772 | 1:50 |
| SHH (5E1) | DSHB | - | 1:20 (TSA amplification) |
| SOX2 | Abcam | ab79351 | 1:100 (TSA amplification) |
| SOX9 | Millipore | AB5535 | 1:300 |
| T1α | Abcam | ab11936 | 1:100 |
