# Supplementary material for: Epithelial heparan sulfate regulates Sonic Hedgehog signaling in lung development
Source: PLoS Genet. 2017 Aug 31;13(8):e1006992. doi: 10.1371/journal.pgen.1006992 (PMC5597256; doi:10.1371/journal.pgen.1006992)
Supplement: S2 Table — (DOCX) [file pgen.1006992.s010.docx]

**S2 Table**

| Primers for qPCR | | | |
| --- | --- | --- | --- |
|  | | Forward | Reverse |
| *Axin2* | | TGACTCTCCTTCCAGATCCCA | TGCCCACACTAGGCTGACA |
| *Arhgef19* | | AGGCCCAGATACCCATATCG | GCCTCTTCGTTATGATACACCTG |
| *Bmp4* | | TTCCTGGTAACCGAATGCTGA | CCTGAATCTCGGCGACTTTTT |
| *Celsr1* | | TCGCTGACTTCGGTGCTTG | TTACCAGCTCTACCCAAACGG |
| *Cdh1* | | CAGGTCTCCTCATGGCTTTGC | CTTCCGAAAAGAAGGCTGTCC |
| *Dusp6* | | ATAGATACGCTCAGACCCGTG | ATCAGCAGAAGCCGTTCGTT |
| *Etv4* | | CGGAGGATGAAAGGCGGATAC | TCTTGGAAGTGACTGAGGTCC |
| *Etv5* | | TCAGTCTGATAACTTGGTGCTTC | GGCTTCCTATCGTAGGCACAA |
| *Fgf9* | | CCCAACGGTACTATCCAGGGA | AGGCCCACTGCTATACTGATAAA |
| *Fgf10* | | TTTGGTGTCTTCGTTCCCTGT | TAGCTCCGCACATGCCTTC |
| *Fgfr2* | | AATCTCCCAACCAGAAGCGTA | CTCCCCAATAAGCACTGTCCT |
| *Gapdh* | | GCCCTTCCACAATGCCAAAG | ATCACCATCTTCCAGGAGCGAG |
| *Gli1* | | CCAAGCCAACTTTATGTCAGGG | AGCCCGCTTCTTTGTTAATTTGA |
| *Hhip1* | | TGAAGATGCTCTCGTTTAAGCTG | CCACCACACAGGATCTCTCC |
| *Ptch1* | | AAAGAACTGCGGCAAGTTTTTG | CTTCTCCTATCTTCTGACGGGT |
| *Shh* | | AAAGCTGACCCCTTTAGCCTA | TTCGGAGTTTCTTGTGATCTTCC |
| *Spry2* | | TCCAAGAGATGCCCTTACCCA | GCAGACCGTGGAGTCTTTCA |
| *Wnt2* | | CTCGGTGGAATCTGGCTCTG | CACATTGTCACACATCACCCT |
| *Wnt7b* | | TTTGGCGTCCTCTACGTGAAG | CCCCGATCACAATGATGGCA |
| *Vangl2* | | ACTCGGGCTATTCCTACAAGT | TGATTTATCTCCACGACTCCCAT |
| *Vimentin* | | CGTCCACACGCACCTACAG | GGGGGATGAGGAATAGAGGCT |
|  | |  |  |
| Primers for riboprobe synthesis | | | |
|  | Forward | | Reverse |
| *Fgf10* | AATTAACCCTCACTAAAGGGAGACTTCCTCCTCGTCCTTCT | | TAATACGACTCACTATAGGGAGAGTACTGCATCCACCAACA |
| *Shh* | AATTAACCCTCACTAAAGGGAGACCAGCGGCAGATATGAAG | | TAATACGACTCACTATAGGGAGACCAGGAAGGTGAGGAAGT |
| *Gli1* | AATTAACCCTCACTAAAGGGAGACCTCCTCCTCTCATTCCA | | TAATACGACTCACTATAGGGAGAAGAGTCCAGAGCGTTACA |
| *Ptch1* | AATTAACCCTCACTAAAGGGAGACTTGGTGTTGGTGTGGAT | | TAATACGACTCACTATAGGGAGAACTGTGAGGCTCTGTGTA |
| *Axin2* | AATTAACCCTCACTAAAGGGA  CAGATCGGCTCGGTCATGTT | | TAATACGACTCACTATAGGGAGACGGATTGACTGGGTCGCTTCT |
| *Wnt2* | AATTAACCCTCACTAAAGGGA  TAGATGCCAAGGAGAGGAA | | TAATACGACTCACTATAGGGAGACTGATACAGTAGTCTGGAGAAT |
| *Wnt7b* | AATTAACCCTCACTAAAGGGA GCCTTCACCTATGCCATC | | TAATACGACTCACTATAGGGAGACTGCGTTGTACTTCTCCTT |
